# Degradation and Detoxification of Chlorophenols with Different Structure by LAC-4 Laccase Purified from White-Rot Fungus *Ganoderma lucidum*

**DOI:** 10.3390/ijerph19138150

**Published:** 2022-07-02

**Authors:** Wei Deng, Wei Zhao, Yang Yang

**Affiliations:** Hubei Key Laboratory of Genetic Regulation and Integrative Biology, School of Life Sciences, Central China Normal University, Wuhan 430079, China; dengw112@163.com (W.D.); zhaow1232022@163.com (W.Z.)

**Keywords:** laccase, chlorophenol, degradation, detoxification, phytotoxicity, degradation mechanisms

## Abstract

A laccase named LAC-4 was purified from *Ganoderma lucidum*. Firstly, the enzymatic properties of purified LAC-4 laccase, and the degradation of three chlorophenol pollutants 2,6-dichlorophenol (2,6-DCP), 2,3,6-trichlorophenol (2,3,6-TCP) and 3-chlorophenol (3-CP) by LAC-4 were systematically studied. LAC-4 had a strong ability for 2,6-DCP and 2,3,6-TCP degradation. The degradation ability of LAC-4 to 3-CP was significantly lower than that of 2,6-DCP and 2,3,6-TCP. LAC-4 also had a good degradation effect on the chlorophenol mixture (2,6-DCP + 2,3,6-TCP). The results of kinetics of degradation of chlorophenols by LAC-4 suggested that the affinity of LAC-4 for 2,6-DCP was higher than 2,3,6-TCP. The catalytic efficiency and the catalytic rate of LAC-4 on 2,6-DCP were also significantly higher than 2,3,6-TCP. During degradation of 2,6-DCP and 2,3,6-TCP, LAC-4 had a strong tolerance for high concentrations of different metal salts (such as MnSO_4_, ZnSO_4_, Na_2_SO_4_, MgSO_4_, CuSO_4_, K_2_SO_4_) and organic solvents (such as ethylene glycol and glycerol). Next, detoxification of chlorophenols by LAC-4 was also systematically explored. LAC-4 treatment had a strong detoxification ability and a good detoxification effect on the phytotoxicity of individual chlorophenols (2,6-DCP, 2,3,6-TCP) and chlorophenol mixtures (2,6-DCP + 2,3,6-TCP). The phytotoxicities of 2,6-DCP, 2,3,6-TCP and chlorophenol mixtures (2,6-DCP + 2,3,6-TCP) treated with LAC-4 were considerably reduced or eliminated. Finally, we focused on the degradation mechanisms and pathways of 2,6-DCP and 2,3,6-TCP degradation by LAC-4. The putative transformation pathway of 2,6-DCP and 2,3,6-TCP catalyzed by laccase was revealed for the first time. The free radicals formed by LAC-4 oxidation of 2,6-DCP and 2,3,6-TCP produced dimers through polymerization. LAC-4 catalyzed the polymerization of 2,6-DCP and 2,3,6-TCP, forming dimer products. LAC-4 catalyzed 2,6-DCP into two main products: 2,6-dichloro-4-(2,6-dichlorophenoxy) phenol and 3,3′,5,5′-tetrachloro-4,4′-dihydroxybiphenyl. LAC-4 catalyzed 2,3,6-TCP into two main products: 2,3,6-trichloro-4-(2,3,6-trichlorophenoxy) phenol and 2,2′,3,3′,5,5′-hexachloro-[1,1′-biphenyl]-4,4′-diol.

## 1. Introduction

Laccase is a multi-copper oxidase that has an active center containing four copper atoms. Laccase can oxidize a substrate into free radicals by extracting electrons from it. Additional non-enzymatic secondary reactions could occur due to the instability of free radicals. Meanwhile, the oxygen molecule is reduced to water as the electron acceptor [1,2,3]. Because of its low substrate specificity, a wide range of substrates and unique degradation function, laccase is widely used in industrial and environmental biotechnology fields. Laccase has advantages over traditional approaches in the degradation of pollutants: the rate of degradation of pollutants is fast and efficient, the cost is low, and the operation is simple. The high efficiency and lack of secondary pollution of laccase in degrading pollutants make it attractive and valuable in environmental pollutant treatment, including wastewater treatment, soil remediation, and industrial dye bleaching [4,5,6,7,8,9].

Chlorophenol is a type of phenol organic compound containing one or more covalently bound chlorine atoms. Chlorophenol compounds are widely used in chemical production and other fields, including wood preservatives, fungicides and herbicides. 2,6-dichlorophenol is an important chemical raw material widely used in chemicals, medicine, and pesticides [10,11]. Although chlorophenol compounds have a wide range of uses in industrial production and human activities, they are persistent, stubborn and highly toxic compounds that are widely present in the environment and water bodies, showing high acute toxicity and genotoxicity [12,13,14,15,16]. Chlorophenol in the human body will damage the human nervous system and respiratory system, causing endocrine disorders. Chlorophenol also has potential carcinogenic effects [17,18]. The International Agency for Research on Cancer has classified chlorophenol as a category 2B carcinogen (a possible carcinogen). Based on the above concerns and the high persistence, bioaccumulation and low biodegradability of chlorophenol in the environment, chlorophenol is classified as a primary pollutant and toxin by the US Environmental Protection Agency [11,12]. Taken together, the heavy use of chlorophenols in modern industry has caused them to be discharged into the natural environment through various channels, causing serious threats and harm to the ecological environment and human health. The control of chlorophenol pollution has become an urgent need to solve important environmental problems.

In contrast to peroxidase, laccase, which does not require H_2_O_2_ to participate in the catalytic process, has attracted much attention in the treatment of chlorophenol pollutants [19,20,21,22,23,24,25]. The ability of laccase to degrade chlorophenol depends on the number of chlorine atoms and their position in the phenol structure. Laccase cannot readily remove meta-chlorophenol [26]. Gaitan et al. evaluated the toxicity and degradation of a chlorophenol mixture by the laccase produced by *Trametes pubescens* [27]. Zhang et al. used laccase from *Coriolus versicolor* to catalyze the degradation of 2-chlorophenol, 4-chlorophenol and 2,4-dichlorophenol. The effects of reaction time, pH value, temperature, and laccase and chlorophenol concentration on the degradation efficiency were studied. The results showed that laccase had the strongest catalytic ability for 2,4-dichlorophenol [21]. Previous research suggested that the ability of laccase secreted by *Coriolus versicolor* to degrade mono-chlorophenol ranks para > ortho > meta, meta-substituted phenol is relatively difficult to degrade [28]. Kadhim et al. found that different positions of chlorine substituents could affect the removal efficiency of laccase, and phenolic compounds with ortho- and para-chlorine substitution were more susceptible to laccase attack than meta-substituted compounds [29]. Under laccase-catalyzed conditions, the dechlorination of chlorophenol occurs due to the free radical mechanism of the oxidative coupling reaction. Harsh pH conditions may lead to insufficient free radical production and the reduction in chlorophenol dechlorination [30]. Previous studies indicated that laccase catalyzes 2,4-dichlorophenol to form 4 free radicals. These 4 free radicals can form 10 possible oxidative coupling reactions, 7 of which involve the release of chloride ions [30,31]. Leontievsky et al. found that laccase from *Coriolus versicolor* oxidized 2,4,6-trichlorophenol to 2,6-dichloro-1,4-benzoquinone and 2,6-dichloro-1,4-dihydroxybenzene, as well as low-molecular-weight polymers with a molecular weight of 360 kDa and polymers with a molecular weight higher than 1000 kDa. The first two are the main products of laccase oxidation of 2,4,6-trichlorophenol [32]. As a highly toxic environmental pollutant, the elimination of the toxicity of chlorophenol is key to the treatment of chlorophenol pollutants. Yin et al. evaluated the toxicity of mono-chlorophenol and its degradation products using *E. coli* expressing GFP [33], and Gaitan et al. determined the toxicity of chlorophenol and its degradation products using the luminescence degree of luminescent bacteria [27]. Laccase is a green and environmentally friendly enzyme catalyst, which has great application values and potential in the field of chlorophenol pollutant treatment.

The existing research on laccase degradation of chlorophenol mainly focuses on the degradation ability and degradation efficiency, while studies on the degradation mechanisms and degradation pathways through which laccase degrades chlorophenol and the detoxification effect of laccase on chlorophenol are lacking and not systematic enough. The degradation mechanisms, conversion pathways and detoxification effects of laccase on two important chlorophenols 2,6-dichlorophenol (2,6-DCP) and 2,3,6-trichlorophenol (2,3,6-TCP) are unknown and not explored. To date, only a few studies have identified the products of laccase catalysis of chlorophenols and have evaluated the toxicity of the degradation products. The detoxification effect of laccase on the phytotoxicity of chlorophenols was not studied in the previous research [27,33]. An in-depth investigation of the degradation pathways through which laccase degrades chlorophenol and the detoxification effects of laccase on chlorophenol is significantly important for eliminating the damage caused by chlorophenol pollutants to the environment and human health.

In this study, a laccase named LAC-4 was successfully purified from *Ganoderma lucidum*. We systematically studied the enzymatic properties of LAC-4 laccase and explored the degradation ability of LAC-4 on three chlorophenol pollutants, namely, 2,6-dichlorophenol (2,6-DCP) and 2,3,6-trichlorophenol (2,3,6-TCP) and 3-chlorophenol (3-CP). We assessed the tolerance of LAC-4 for different metal salts and organic solvents during the degradation process. Based on the germination rates and seedling growth behaviors of three plant seeds (mung bean, wheat, and rice), we evaluated the detoxification abilities of LAC-4 on the phytotoxicities of 2,6-DCP and 2,3,6-TCP. Moreover, this work focused on the mechanisms and pathways of 2,6-DCP and 2,3,6-TCP degradation by LAC-4, and we revealed the degradation pathway of LAC-4 laccase, which catalyzed the conversion of 2,6-DCP and 2,3,6-TCP. Our results have a positive role in promoting the better application of LAC-4 laccase in the treatment of chlorophenol wastewater or the remediation of an environment contaminated by chlorophenol.

## 2. Materials and Methods

### 2.1. Strain and Medium

*Ganoderma lucidum* was preserved in Central China Normal University, Wuhan, China. The GYP medium was used for the production of laccase. The components of the GYP medium are: glucose 20 g/L, yeast extract 5 g/L, peptone 5 g/L, MgSO_4_·7H_2_O 1 g/L, CuSO_4_·5H_2_O 0.002 g/L.

### 2.2. Purification of LAC-4 Laccase

When the laccase activity in the cell culture reached the highest level, the gauze was filtered to remove the mycelium, and the filtrate was collected. The filtrate was frozen at −80 °C overnight, thawed at room temperature and filtered with gauze to remove the precipitated polysaccharide, after which the filtrate was collected again. Ammonium sulfate with a saturation of 80% was added to the filtrate and allowed to settle overnight at 4 °C. The crude enzyme solution precipitated by ammonium sulfate was centrifuged at 8000 rpm for 15 min, the supernatant was discarded, and the precipitate was collected. The precipitate was dissolved with 20 mM pH 6.5 citrate buffer and centrifuged at 8000 rpm for 15 min to remove undissolved impurities. Then, the collected supernatant was packed into a dialysis bag and dialyzed for 24 h (cutoff of 14 kDa). The concentrated enzyme solution was filtered through a 0.22 μm filter, and the sample was subjected to anion exchange chromatography. After sample loading, the unbound heterogenous protein was eluted with 100 mL of CPBS, and the sample was eluted with a linear gradient of 0–1 M NaCl solution (prepared with CPBS) for 100 min. The eluent with an absorption peak at 280 nm was collected in separate tubes (3 mL per tube) to detect laccase activity. The eluate containing laccase was collected in separate tubes, and the enzyme solutions with high enzyme activities were combined for dialysis and concentration. Next, the treated enzyme solution was loaded for hydrophobic chromatography. After sample loading, the unbound contaminating protein was eluted with 100 mL of ammonium sulfate solution. Then, the sample was eluted with a linear gradient of 1–0 M ammonium sulfate solution for 100 min. The eluent with high laccase activity was collected as described above and was then concentrated by dialysis.

### 2.3. Study on Enzymatic Properties of Purified LAC-4

#### 2.3.1. Kinetic Studies on LAC-4 Laccase

Three substrates (2,2′-azino-bis(3-ethyl-benzthiazoline-6-sulphonic acid (ABTS), guaiacol and 2,6-dimethoxyphenol) were selected to determine the kinetics of LAC-4 laccase. ABTS: the concentration is 0.01–0.1 mM and the measurement wavelength is 420 nm. 2,6-dimethoxyphenol: the concentration is 0.01–0.06 mM and the measurement wavelength is 470 nm. Guaiacol: the concentration is 0.1–1.0 mM and the measurement wavelength is 465 nm. The above reaction system is 2 mL, and the amount of LAC-4 laccase is 0.01 U/mL. The reaction was carried out in 50 mM pH 5.0 acetate buffer at 30 °C. Three replicates were set for each experimental group. The kinetic parameters of LAC-4 laccase were calculated by non-linear regression model based on the Michaelis–Menten equation.

#### 2.3.2. Optimal Reaction Temperature and Stability of Laccase at Different Temperatures

Laccase activity was measured at different temperatures (20–85 °C, at intervals of 5 °C), where the highest laccase activity value was set to 100%, and relative laccase activity at each temperature was calculated. Then, the optimal enzymatic reaction temperature for LAC-4 was determined. The reaction was carried out in 50 mM acetate buffer at a pH of 5.0, and the selected substrate was ABTS with a final concentration of 1 mmol/L. In addition, 0.01 U/mL of LAC-4 was used in the reaction system.

The enzyme solution was assessed at different temperatures, and laccase activity was measured at different times (0 h, 4 h, 12 h, 1 d, 3 d, 5 d, 7 d). Laccase activity measured at 0 h was set to 100%, and relative laccase activity at different times was calculated for each temperature condition to determine the stability of LAC-4 at different temperatures. The reaction was carried out in 50 mM acetate buffer at a pH of 5.0, and the selected substrate was ABTS with a final concentration of 1 mmol/L. 0.01 U/mL of LAC-4 was used in the reaction system.

#### 2.3.3. Optimal Reaction pH and Stability of Laccase at Different pH

Laccase activity was measured at different pH levels (pH 1.0–7.0, at intervals of 0.5), where the highest laccase activity value was set to 100%, and relative laccase activity at each pH was calculated. Then, the optimal enzymatic reaction pH for LAC-4 was determined.

The stability of LAC-4 laccase at different pH (1.0–13.0) was determined under the condition of 30 °C. The enzyme solution was assessed at different pH, and laccase activity was measured at different times (0 h, 4 h, 12 h, 1 d, 3 d, 5 d, 7 d). Laccase activity measured at 0 h was set to 100%, and relative laccase activity at different times was calculated for each pH condition to determine the stability of LAC-4 at different pH.

#### 2.3.4. Effect of Inhibitors on the Activity of LAC-4

DTT (0.0005, 0.005, 0.05 mM), EDTA-2Na (0.5, 5, 10, 25, 50, 100, 200 and 400 mM), NaN_3_ (0.0005, 0.005 and 0.05 mM), SDS (100, 200 and 400 mM) and mercaptoethanol (0.005, 0.05 and 0.5 mM) were added to the laccase reaction system, and laccase activity was measured after the sample was thoroughly mixed. Enzyme activity was measured by adding an equal volume of acetate buffer, which was used as the control and set to 100%, and the relative activity of LAC-4 under different concentrations of inhibitors was calculated for various inhibitors. The reaction was carried out in 50 mM acetate buffer at a pH of 5.0, and the selected substrate was ABTS with a final concentration of 1 mmol/L. 0.01 U/mL of LAC-4 was used in the reaction system. The reaction temperature is 30 °C.

#### 2.3.5. Effect of Different Metal Ions and Organic Solvents on the Activity of LAC-4

Different metal salts (Al_2_(SO_4_)_3_, AlCl_3_, CaCl_2_, CdCl_2_, CoCl_2_, NiCl, LiCl, CuSO_4_, CuCl_2_, K_2_SO_4_, KCl, MgSO_4_, MgCl_2_, MnSO_4_, MnCl_2_, Na_2_SO_4_, NaCl, ZnSO_4_, ZnCl_2_) (final concentration: 10–800 mM) were added to the laccase reaction system, and laccase activity was measured after the sample was thoroughly mixed. Enzyme activity was measured by adding an equal volume of acetate buffer, which was used as the control and set to 100%, and the relative activity of LAC-4 under different concentrations of metal ions was calculated for various metal ions.

Different organic solvents (methanol, ethanol, ethylene glycol, propylene glycol, glycerol, isopropanol, butanediol, acetonitrile, acetone, DMSO, DMF) (final concentration: 10–50%, *v*/*v*) were added to the laccase reaction system, and laccase activity was measured after the sample was thoroughly mixed. Enzyme activity was measured by adding an equal volume of acetate buffer, which was used as the control and set to 100%, and the relative activity of LAC-4 under different concentrations of organic solvents was calculated for various organic solvents.

#### 2.3.6. Effect of Different Metal Ions and Organic Solvents on the Stability of LAC-4

LAC-4 laccase was incubated with different metal ions (concentration was 10, 50, 100 mM) at 30 °C to determine laccase activity at different time points (0 h, 4 h, 12 h, 1 d, 3 d, 5 d, 7 d), and a sample incubated with laccase and acetate buffer at 30 °C was used as the control (no metal ions were added). The laccase activity measured at 0 h was set to 100%, and then the relative laccase activity at each time point was calculated.

LAC-4 laccase was incubated with different organic solvents (concentration was 10%, 20%, 50%, *v*/*v*) at 30 °C to determine laccase activity at different time points (0 h, 4 h, 12 h, 1 d, 3 d, 5 d, 7 d), and a sample incubated with laccase and acetate buffer at 30 °C was used as the control (no organic solvents were added). The laccase activity measured at 0 h was set to 100%, and then the relative laccase activity at each time point was calculated.

### 2.4. Degradation of Different Concentrations of Chlorophenols by LAC-4

The degradation of different chlorophenols (2,6-dichlorophenol, 2,3,6-trichlorophenol, 3-chlorophenol) by LAC-4 was performed in 50 mM acetate buffer (pH 5.0), and the reaction was carried out at 30 °C for 12 h. 2 mL reaction system included: 1 U/mL LAC-4, different concentrations of chlorophenol (100, 200, 400, 600, 800, 1000, 2000 mg/L), 50 mM pH 5.0 acetate buffer. An equal volume of chromatographic grade ethyl acetate was added to the degradation product for extraction. After centrifugation, the upper organic phase was obtained, filtered through a 0.22 μm microporous filter, and the sample was filtered for HPLC detection.

The high-performance liquid chromatography (HPLC) detection conditions were as follows: the mobile phase was composed of a water-methanol mixture (*v*/*v*), the flow rate was 0.7 mL/min, the detection wavelength of the ultraviolet (UV) detector was 215 nm, and the column temperature was 35 °C. Chlorophenol concentration was calculated according to the plotted standard curve of chlorophenol. The calculation formula of chlorophenol degradation efficiency is as follows: degradation efficiency (%) = (A0 − Ai)/A0 × 100%; Where A0 is the initial concentration and Ai is the concentration after degradation.

In addition, equal concentrations of 2,6-DCP and 2,3,6-TCP (100 + 100 mg/L, 200 + 200 mg/L, 400 + 400 mg/L) were added to the reaction system to measure the degradation ability of LAC-4 on the mixed chlorophenols.

### 2.5. Study on the Kinetics of Degradation of Two Chlorophenols by LAC-4

The kinetics of degradation of the two chlorophenols by LAC-4 were assessed using the Michaelis-Menten equation. The 2 mL reaction system consisted of chlorophenol (0.1, 0.2, 0.4, 0.6, 0.8, 1, 2, 3, 5 and 8 mM), LAC-4 laccase (1 U/mL), and 50 mM acetate buffer (pH 5.0), and the reaction was carried out at 30 °C for either 20 min (2,6-dichlorophenol, 2,6-DCP) or 30 min (2,3,6-trichlorophenol, 2,3,6-TCP). The kinetic parameters for the two chlorophenols degraded by LAC-4 were calculated using the Lineweaver-Burk double reciprocal plot method.

The reaction rate constant k value was calculated using the ln c-t plot method. The degradation system contained 50 mM of acetate buffer (pH 5.0), 100 mg/L of chlorophenol, 0.5 U/mL of LAC-4, and the degradation reaction was carried out at 30 °C. Samples were collected at different times, the remaining substrate concentration in the HPLC detection system was recorded as c, and the natural logarithm of c was calculated, ln c. The graph of ln c versus time (t) was plotted, where the ln c–t fitting curve was a straight line, and the negative value of the slope of the straight line was the reaction rate constant, k.

### 2.6. The Effects of Metal Salts and Organic Solvents on the Degradation of Chlorophenols by LAC-4

The degradation reaction system was 2 mL, including 200 mg/L 2,6-DCP or 2,3,6-TCP, LAC-4 laccase (1 U/mL), different concentrations of metal salts or organic solvents, 50 mM pH 5.0 acetate buffer. The degradation system without any metal salts and organic solvents was used as the control. After the reaction system was evenly mixed, it was allowed to rest at 30 °C and the reaction was carried out for 12 h. HPLC was then used to determine the remaining chlorophenol content. The HPLC detection method and the calculation method of chlorophenol degradation efficiency were as described in Section 2.4.

### 2.7. Phytotoxicity Testing of Degradation Products

The germination of three plant seeds was used as an index to detect the phytotoxicity of chlorophenol before and after degradation. Rice seeds were washed with distilled water and soaked at 30 °C for 12 h, allowing them to germinate. Rice seeds with full grains and signs of germination were selected for the experiment. A piece of Whatman filter paper was placed in each dish, 20 rice seeds with full grains were randomly placed on the filter paper, and 4 mL of distilled water (blank control group), undegraded 2,6-DCP (undegraded experimental group) or 2,6-DCP (degradation product of the experimental group) degraded by laccase was added. The filter paper was soaked completely, and the dishes were placed in a 28 °C biochemical incubator for dark cultivation for 120 h. To ensure that the seeds had sufficient oxygen, the lid of the dish was opened every 24 h for 5 min. After 120 h of cultivation, the root length and shoot length of the rice seeds were measured, and the germination rate was calculated: germination rate = (number of seeds germinated/total number of seeds) × 100%.

### 2.8. Determination of Chloride Ion Concentration by Silver Nitrate Turbidimetry: Dechlorination Study

The samples of 2,6-DCP and 2,3,6-TCP degraded by LAC-4 were extracted with ethyl acetate, and 2 mL of the aqueous phase sample was added to 10 mL of nitric acid, 2 mL of absolute ethanol and 2 mL of 2% silver nitrate solution, and the volume was adjusted to 50 mL. Shaking was performed for 20 min, and the absorbance was measured at 460 nm. The chloride ion concentration was calculated using a standard curve.

### 2.9. HPLC Detection of Chlorophenol

An equal volume of chromatographic grade ethyl acetate was added to the degradation product for extraction. After centrifugation, the upper organic phase was obtained, filtered through a 0.22 μm microporous filter, and the sample was filtered for HPLC detection. The elution procedure used was as follows: 0–15 min, the methanol gradient was 70–100% (*v*/*v*); 15–18 min, the methanol was kept at 100%; 18–32 min, the methanol gradient was 70–100%; 32–39 min, the methanol concentration returned to the initial condition (70%) for 7 min.

### 2.10. GC-MS Detection of LAC-4 Degradation of the 2,6-Dichlorophenol Intermediate

The dosage of LAC-4 was 1 U/mL, the concentration of 2,6-DCP was 600 mg/L, and the degradation reaction system volume was 2 mL. After the preparation of the degradation system, it was placed at 30 °C for reaction. The system without enzyme solution was taken as the 0 h sample, and samples were collected at 0.5 h, 3 h and 12 h for extraction. Four degradation samples were measured in parallel at each time point. At each time point, the degraded sample was extracted with ethyl acetate multiple times to ensure full extraction, and then the extracted organic phase was separated and concentrated. The extracted sample was filtered using a 0.22 μm microporous filter and was then subjected to GC-MS detection. Gas chromatography-mass spectrometry instrument: Varian450-GC, 320-MS; Chromatographic column: VF-5 chromatographic column (30 mm × 0.25 mm). The GC-MS detection conditions were as follows: the initial column temperature was 50 °C for 3 min; the temperature was linearly increased to 290 °C at a rate of 20 °C/min and was maintained for 10 min. The inlet temperature was 280 °C, and the ion source EI temperature was 200 °C.

### 2.11. Statistical Analysis

All measurements were repeated three times. The data are expressed as the mean ± standard deviation, and a t-test or ANOVA was performed to evaluate the significant differences between means. All data were analyzed using Graphpad Prism 5.01 software for the *t*-test of the difference between the two groups of means, and significant differences between two groups of means were evaluated using the *p*-value. A *p*-value < 0.01 indicated that the difference was very significant (presented as **). A *p*-value < 0.05 indicated that the difference was significant (presented as *).

## 3. Results

### 3.1. Purification of LAC-4 Laccase from Ganoderma lucidum

**Figure 1:**
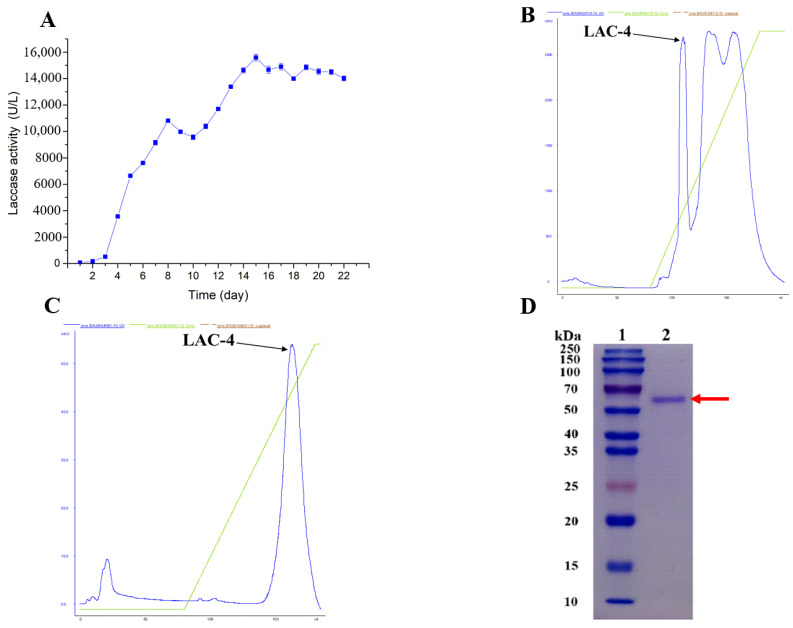
Purification of LAC-4 laccase from white-rot fungus *Ganoderma lucidum*. (**A**) Change curve of laccase production by *Ganoderma lucidum* in GYP medium. CuSO_4_ was added on the third day of incubation to induce laccase expression. (**B**) The chromatographic profile of LAC-4 (anion chromatography). (**C**) The chromatographic profile of LAC-4 (hydrophobic interaction chromatography). (**D**) Detection of purified LAC-4 laccase by SDS-PAGE electrophoresis. Lane 1: protein molecular mass marker; Lane 2: purified LAC-4 laccase.

The fungal strain was incubated using a glucose yeast extract peptone (GYP) enzyme production medium, and sample was collected daily to determine the laccase activity in the medium. The results are shown in Figure 1. CuSO_4_ was added on the third day of incubation to induce laccase expression in the fungal strain. After adding CuSO_4_, laccase activity increased rapidly, and it was highest on the fifteenth day, while laccase activity was 15,580 U/L (Figure 1A). The crude laccase enzyme solution was then collected, and the purified LAC-4 laccase protein was finally obtained using ammonium sulfate precipitation, anion chromatography, and hydrophobic interaction chromatography (Figure 1B,C). The sodium dodecyl sulfate-polyacrylamide gel electrophoresis (SDS-PAGE) results showed only one protein band, indicating that the purified LAC-4 reached electrophoretic purity, and the molecular weight of LAC-4 laccase was approximately 56 kDa (Figure 1D).

### 3.2. Study on the Enzymatic Properties of Purified LAC-4 Laccase

#### 3.2.1. Kinetic Studies on the Purified LAC-4

As shown in Table 1, the Km values of LAC-4 on ABTS, 2,6-dimethoxyphenol (2,6-DMP), and guaiacol were 0.0746, 0.8034, and 0.0971, respectively. These results indicated that LAC-4 had the highest affinity for ABTS, followed by guaiacol, and the lowest affinity for 2,6-DMP. For the enzymatic reaction with LAC-4, when ABTS was used as the substrate, the maximum reaction rate was 3.988 × 10^−7^ mM/s. In the catalytic reactions of 2,6-DMP and guaiacol, the maximum reaction rates were 1.085 × 10^−7^ and 1.906 × 10^−7^ mM/s, respectively. Compared to 2,6-DMP and guaiacol, LAC-4 had the fastest reaction rate to ABTS and highest affinity for ABTS, indicating that the most suitable substrate for LAC-4 was ABTS. The Kcat/Km value also suggested that LAC-4 had the highest catalytic efficiency for ABTS and the lowest catalytic efficiency for 2,6-DMP (Table 1).

#### 3.2.2. Effect of Temperature on the Activity and Stability of LAC-4

**Figure 2:**
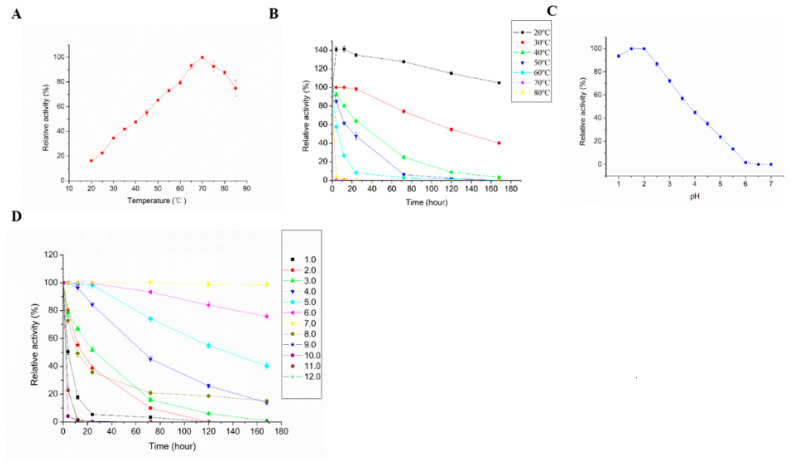

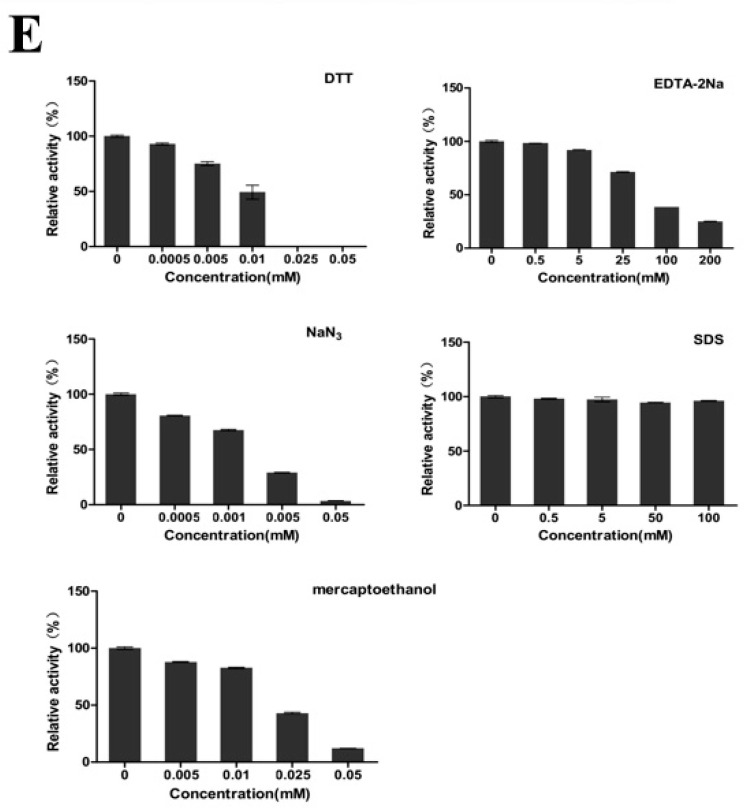
Enzymatic properties of purified LAC-4 laccase. (**A**) Effects of different temperatures on LAC-4 activity. (**B**) Effects of different temperatures on LAC-4 stability. (**C**) Effects of different pH on LAC-4 activity. (**D**) LAC-4 stability at different pH values. (**E**) Effects of different inhibitors (SDS, EDTA-2Na, DTT, NaN_3_, and mercaptoethanol) on LAC-4 laccase activity.

The effects of different temperatures on LAC-4 activity are shown in Figure 2A, indicating that the optimal reaction temperature for LAC-4 was 70 °C. The enzyme activity gradually increased with increasing temperature, and the highest laccase activity was observed at 70 °C. When the temperature continued to increase, enzymatic activity declined. The effects of different temperatures on LAC-4 stability are shown in Figure 2B. LAC-4 had good stability at 20 °C and 30 °C. When LAC-4 was incubated at 20 °C for 7 days, no significant changes in activity were observed. When the temperature increased to 30 °C, the remaining laccase activity was 40% after 7 days. However, as the temperature further increased, LAC-4 stability decreased considerably (Figure 2B).

#### 3.2.3. Effect of pH on the Activity and Stability of LAC-4

The effects of different pH values on LAC-4 laccase activity are shown in Figure 2C, showing that the optimal reaction pH for LAC-4 was 2. When the pH value was in the range of 1–2, LAC-4 exhibited the highest activity, and when the pH value was in the range of 2–6, activity decreased as the pH increased. When the pH reached 6, the activity of LAC-4 was only 1.68% (Figure 2C). LAC-4 stability at different pH values is shown in Figure 2D, indicating that LAC-4 had the best stability under neutral pH 7.0 conditions, and activity remained above 98% after 7 days. LAC-4 also exhibited good stability at pH 6.0, and LAC-4 activity remained above 80% after 7 days. Under acidic conditions, the stability of LAC-4 gradually decreased as the pH decreased. Thus, when the pH was 1.0, the enzymatic activity of LAC-4 declined to 50% after 4 h, and after 24 h, the enzymatic activity was only 5%. LAC-4 had poor stability under alkaline conditions. When the pH values were 9.0 and 10.0, the remaining activities after 4 h were only 4.05% and 4.24%, respectively. Thus, LAC-4 laccase stability under neutral and acidic conditions was considerably higher than under alkaline conditions (Figure 2D).

#### 3.2.4. Effect of Inhibitors on the Activity of LAC-4

The effects of different inhibitors on LAC-4 laccase activity are shown in Figure 2E. LAC-4 had the strongest tolerance for SDS, and SDS with a concentration of less than 100 mM did not significantly affect the activity of LAC-4. The enzyme also exhibited strong tolerance for ethylenediaminetetraacetic acid disodium salt (EDTA-2Na). LAC-4 still maintained 38.68% and 25.20% activity in 100 and 200 mM EDTA-2Na, respectively. Furthermore, dithiothreitol (DTT), NaN_3_, and mercaptoethanol exhibited strong inhibitory effects on LAC-4 activity, as 0.025 mM DTT could completely inactivate LAC-4, and 0.05 mM NaN_3_ and mercaptoethanol resulted in 3.48% and 12.0% of the remaining LAC-4 activity, respectively (Figure 2E).

#### 3.2.5. Effect of Metal Ions and Organic Solvents on the Activity of LAC-4

**Figure 3:**
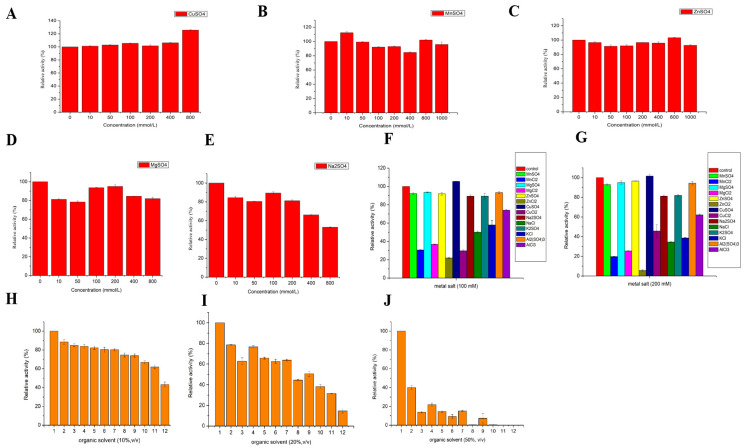
Effects of different metal ions and organic solvents on the LAC-4 laccase activity. (**A**) CuSO_4_, (**B**) MnSO_4_, (**C**) ZnSO_4_, (**D**) MgSO_4_, (**E**) Na_2_SO_4_, (**F**) Comparison of the effects of the metal salt with chloride as the anion and the sulfate ions as the anion on LAC-4 laccase activity (the concentration of metal salt was 100 mM), (**G**) Comparison of the effects of the metal salt with chloride as the anion and the sulfate ions as the anion on LAC-4 laccase activity (the concentration of metal salt was 200 mM), (**H**) Concentration of organic solvent was 10% (*v*/*v*), (**I**) Concentration of organic solvent was 20% (*v*/*v*), (**J**) Concentration of organic solvent was 50% (*v*/*v*). (**H**–**J**): 1: Control, no addition of any organic solvent; 2: Ethylene glycol; 3: Butanediol; 4: Glycerol; 5: Propylene glycol; 6: Ethanol; 7: Methanol; 8: Isopropyl alcohol; 9: Acetonitrile; 10: Acetone; 11: DMSO; 12: DMF.

The effects of different metal ions on the LAC-4 laccase activity are shown in Figure 3, indicating that LAC-4 had strong tolerance for CuSO_4_, ZnSO_4_, MnSO_4_, MgSO_4_, Na_2_SO_4_ (Figure 3A–E). The activity of LAC-4 was retained above 80% in these metal ion solutions, which had concentrations equal to or less than 200 mM. When the concentrations of CuSO_4_, ZnSO_4_, MnSO_4_, MgSO_4_, Na_2_SO_4_ were 800 mM, LAC-4 activity reached 125.64%, 103.30%, 102.06%, 82.01%, 52.99%, respectively. Thus, CuSO_4_ greatly increased the activity of LAC-4 laccase. LAC-4 exhibited the strongest tolerance for MnSO_4_ and ZnSO_4_. Even when the concentrations of MnSO_4_ and ZnSO_4_ increased to 1000 mM, LAC-4 laccase activity still reached 95.82% and 92.73%, respectively, and high activity was maintained. The extremely high concentrations of MnSO_4_ and ZnSO_4_ did not affect the activity of LAC-4 (Figure 3B,C).

As shown in Figure 3F,G, under the same metal cation conditions, the inhibitory effects of the metal salt with chloride as the anion on LAC-4 laccase activity were substantially stronger compared to the sulfate ions as the anion. The metal salt with Cl^−^ as the acid radical ion significantly influenced the activity of LAC-4 laccase. When the concentrations of MnSO_4_, MgSO_4_, ZnSO_4_, CuSO_4_, Na_2_SO_4_, K_2_SO_4_, Al_2_(SO4)_3_ were 100 mM, LAC-4 laccase activity was 92.19%, 93.64%, 91.94%, 105.49%, 89.36%, 89.41%, 93.29%, respectively. When the concentrations of MnCl_2_, MgCl_2_, ZnCl_2_, CuCl_2_, NaCl, KCl, AlCl_3_ were 100 mM, LAC-4 laccase activity decreased to 30.64%, 36.83%, 21.98%, 29.76%, 50.06%, 57.95%, 74.06%, respectively (Figure 3F). Furthermore, a 200 mM concentration of ZnSO_4_ had basically no effect on laccase activity, and LAC-4 laccase activity reached 96.58%. However, 200 mM ZnCl_2_ strongly inhibited LAC-4 laccase activity, and enzymatic activity was only 5.68% (Figure 3G).

The effects of different organic solvents on LAC-4 laccase activity are shown in Figure 3. Among the 11 organic solvents, LAC-4 showed strong tolerance for ethylene glycol, glycerol, and 1,2-propanediol. With an organic solvent concentration of 10% (*v*/*v*), laccase activity remained above 80%, and when the concentration increased to 20% (*v*/*v*), LAC-4 activity still remained above 60% (Figure 3H,I). The tolerance of LAC-4 for ethylene glycol and glycerol was relatively strong. LAC-4 had the strongest tolerance to ethylene glycol and glycerol. When the concentrations of ethylene glycol and glycerol further increased to 50% (*v*/*v*), LAC-4 laccase activity still reached 40.03% and 21.83%, respectively (Figure 3J). However, dimethyl sulfoxide (DMSO), N,N-dimethylformamide (DMF), isopropanol and acetone showed a relatively strong influence on LAC-4 laccase activity, while 50% of DMF, DMSO, isopropanol and acetone completely eliminated the laccase activity (Figure 3J).

Based on a comparison of the effects of various organic solvents on LAC-4 activity, it was found that the influence of organic solvents on laccase was related to their functional group—alcohol had minimal effect on laccase activity. The more hydroxyl groups available, the more resistant LAC-4 laccase would be. For example, DMSO, DMF, and acetone have much higher effects on LAC-4 activity than glycerol and ethylene glycol. LAC-4 is more active in alcohol solutions containing two or three hydroxyl groups than in alcohol solutions containing only one hydroxyl group. Among methanol, ethanol and isopropanol, which contain the same number of alcoholic hydroxyl groups, isopropanol had the greatest effect on LAC-4 activity, followed by ethanol and methanol. This suggests that the longer the alkane chain in the solution, the more detrimental the effect on the activity of laccase. The same conclusion was reached for three organic solvents containing two alcoholic hydroxyl groups, ethylene glycol, 1,2-propanediol and 1,4-butanediol. Glycerol had a greater effect on LAC-4 activity than ethylene glycol. It may be that the number of alcoholic hydroxyl groups has a greater effect on laccase activity than the alkane chain length. In summary, this study found that the influence of organic solvents on laccase activity depends on many factors; among them, the influence of non-alcoholic substances is greater than that of alcohols; among alcohols, the influence of the number of alcoholic hydroxyl groups is greater than that of the alkane chain length.

#### 3.2.6. Effect of Metal Ions and Organic Solvents on the Stability of LAC-4

**Figure 4:**
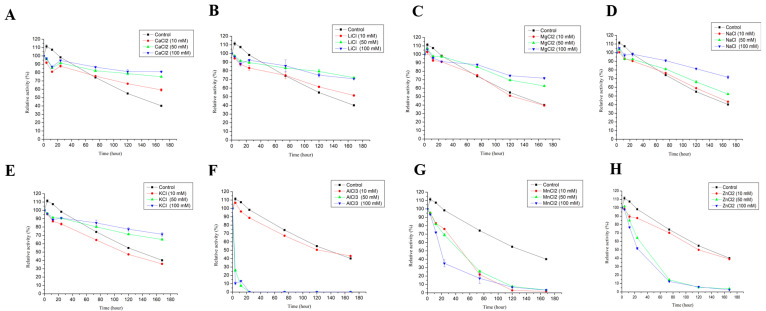
Effects of different metal ions on the stability of LAC-4 laccase. (**A**) CaCl_2_, (**B**) LiCl, (**C**) MgCl_2_, (**D**) NaCl, (**E**) KCl, (**F**) AlCl_3_, (**G**) MnCl_2_, (**H**) ZnCl_2_. Control, no addition of any metal ion.

The effects of different metal ions on the stability of LAC-4 laccase are shown in Figure 4. LAC-4 showed good stability in different concentrations of metal salt solutions, including CaCl_2_, MgCl_2_, NaCl, KCl, LiCl. Compared to the control group, the presence of these metal ions stabilized LAC-4 laccase (stability of LAC-4 laccase was higher) (Figure 4A–E). For example, after LAC-4 was incubated in 10 mM, 50 mM and 100 mM CaCl_2_ solutions for 7 days, the observed laccase activities were 59.23%, 75.00%, and 81.05% of the initial enzymatic activity, respectively. After LAC-4 was incubated in LiCl solutions with different concentrations (10 mM, 50 mM, 100 mM) for 7 days, the laccase activities were 51.51%, 72.03%, and 74.90% of the initial enzyme activity, respectively. However, after LAC-4 was incubated in the buffer without CaCl_2_ and LiCl for 7 days, laccase activity was only 40.17% of the initial enzyme activity. The above results also showed that higher concentrations of CaCl_2_ and LiCl resulted in better LAC-4 laccase stability. The stability of LAC-4 laccase increased with increasing CaCl_2_ and LiCl concentrations (Figure 4A,B).

When metal ions such as AlCl_3_, MnCl_2_, ZnCl_2_ were included in the solution, LAC-4 laccase was less stable than the control. AlCl_3_, MnCl_2_, ZnCl_2_ had a relatively high impact on LAC-4 laccase stability (Figure 4F–H). After LAC-4 was incubated for only 12 h in solutions containing different concentrations of AlCl_3_ (50 mM, 100 mM), laccase activity decreased to 7.59% and 13.11% of the initial enzyme activity, respectively. Furthermore, LAC-4 laccase activity disappeared completely after 24 h of incubation (Figure 4F).

**Figure 5:**
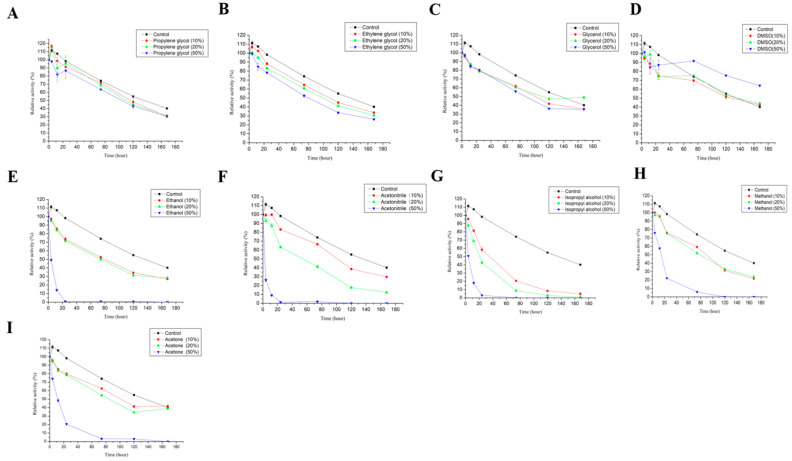
Effects of different organic solvents on the stability of LAC-4 laccase. (**A**) Propylene glycol (**B**) Ethylene glycol (**C**) Glycerol (**D**) DMSO (**E**) Ethanol (**F**) Acetonitrile (**G**) Isopropyl alcohol (**H**) Methanol (**I**) Acetone. Control, no addition of any organic solvent.

The effects of different organic solvents on the stability of LAC-4 laccase are shown in Figure 5. LAC-4 showed good stability in organic solvents such as propylene glycol, ethylene glycol, glycerol, and DMSO, and there were no significant differences in enzyme activity of LAC-4 laccase between the sample incubated in solutions containing different concentrations of propylene glycol, ethylene glycol, glycerol, DMSO, and control samples (Figure 5A–D). In addition, we found that high concentrations of DMSO improved the stability of LAC-4 laccase. After LAC-4 was incubated for 3 d, 5 d, and 7 d in a solution containing 50% (*v*/*v*) DMSO, the remaining laccase activities were still 91.63%, 75.29%, and 64.05% of the initial enzyme activity, respectively. However, laccase activities were only 74.16%, 54.92%, and 40.17% of the initial enzyme activity, respectively, after LAC-4 was incubated for 3 d, 5 d, and 7 d in the buffer without DMSO (Figure 5D). Therefore, compared to the control group (without DMSO), a high concentration of DMSO could substantially improve the stability of LAC-4 laccase.

Ethanol, acetonitrile, isopropanol, methanol, and acetone significantly affected the stability of LAC-4 laccase, and ethanol, acetonitrile, isopropanol, methanol considerably reduced the stability of LAC-4 laccase (Figure 5E–I). In addition, higher concentrations of ethanol and acetonitrile resulted in reduced LAC-4 laccase stability. The higher the concentration of ethanol and acetonitrile, the weaker the stability of LAC-4 laccase (Figure 5E,F). After LAC-4 was incubated for 12 h in a solution containing 50% ethanol or acetonitrile, laccase activity decreased to 14.02% or 8.99% of the initial enzyme activity, and after 24 h of incubation, the LAC-4 laccase activity essentially disappeared (Figure 5E,F).

### 3.3. Study on the Degradation of Chlorophenols with Different Structures by LAC-4

#### 3.3.1. Degradation of 2,6-Dichlorophenol (2,6-DCP), 2,3,6-Trichlorophenol (2,3,6-TCP) and 3-Chlorophenol (3-CP) at Different Concentrations by LAC-4

**Figure 6:**
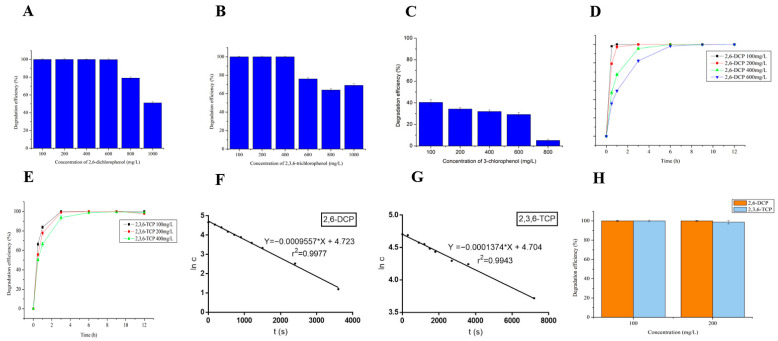
Degradation of chlorophenols with different structures by LAC-4. (**A**) Degradation of different concentrations of 2,6-DCP by LAC-4. (**B**) Degradation of different concentrations of 2,3,6-TCP by LAC-4. (**C**) Degradation of different concentrations of 3-CP by LAC-4. (**D**) The time-course degradation curve for the different concentrations of 2,6-DCP. (**E**) The time-course degradation curve for the different concentrations of 2,3,6-TCP. (**F**) The ln c-t curve for LAC-4 degradation of 2,6-DCP. (**G**) The ln c-t curve for LAC-4 degradation of 2,3,6-TCP. (**H**) Degradation of the 2,6-DCP and 2,3,6-TCP mixture by LAC-4. The concentration of 2,6-DCP and 2,3,6-TCP in the mixture was 100 mg/L, 200 mg/L, respectively.

The degradation of different concentrations of 2,6-DCP, 2,3,6-TCP and 3-CP by LAC-4 are shown in Figure 6A–C, indicating that LAC-4 laccase had a strong ability for 2,6-DCP and 2,3,6-TCP degradation. LAC-4 had the strongest ability for 2,6-DCP degradation, which was completely degraded within 12 h when the concentration of 2,6-DCP was less than 600 mg/L. When the concentration increased to 800, 1000, and 2000 mg/L, the degradation efficiency reached 79.08%, 51.13%, and 16.40%, respectively (Figure 6A). LAC-4 could completely degrade 100 mg/L, 200 mg/L, and 400 mg/L 2,3,6-TCP within 12 h, and the 600, 800, 1000, and 2000 mg/L 2,3,6-TCP degradation efficiency by LAC-4 was 76%, 64.01%, 69.05%, and 5%, respectively (Figure 6B). LAC-4 can also degrade 3-chlorophenol, but the degradation ability of LAC-4 to 3-CP is significantly lower than that of 2,6-DCP and 2,3,6-TCP. Degradation of 3-CP at different concentrations for 12 h are shown in Figure 6C. Except that the degradation efficiency of 100 mg/L reached 40.44%, the degradation efficiencies of other concentrations were all lower than 40%. The 12 h degradation efficiencies of LAC-4 to 100 mg/L, 200 mg/L, 400 mg/L, 600 mg/L and 800 mg/L 3-CP were 40.44%, 34.30%, 31.94%, 29.21% and 5.13%, respectively (Figure 6C).

The time-course degradation curves for the different concentrations of 2,6-DCP and 2,3,6-TCP are shown in Figure 6D,E, indicating that LAC-4 could degrade 2,6-DCP very quickly. As shown in Figure 6D, the 0.5 h 100 mg/L 2,6-DCP degradation efficiency by LAC-4 reached 98.08%, and the 1 h 200 mg/L 2,6-DCP degradation rate reached 97.33%. LAC-4 could completely degrade 100 mg/L 2,6-DCP within 0.5 h. The degradation rate decreased with the increase in 2,6-DCP concentration. The 3 h 400 and 600 mg/L 2,6-DCP degradation efficiency by LAC-4 reached 95.56% and 82.18%, respectively, 6 h degradation efficiency reached 99.89% and 98.20% (Figure 6D). LAC-4 could also quickly degrade 2,3,6-TCP. As shown in Figure 6E, 0.5 h 100 mg/L, 200 mg/L, and 400 mg/L 2,3,6-TCP degradation efficiencies were 66.33%, 55.58%, and 50.25%, respectively. In addition, the 1 h degradation efficiencies were 83.80%, 77.80%, and 66.38%, respectively, and the 3 h degradation efficiencies reached 100%, 99.11%, and 93.6%, respectively (Figure 6E). LAC-4 could completely degrade 2,6-DCP and 2,3,6-TCP at different concentrations within 6 h, and the degradation of 2,6-DCP by LAC-4 was faster than 2,3,6-TCP.

#### 3.3.2. Kinetics of Degradation of Chlorophenols by LAC-4

The kinetics of degradation of the two chlorophenols by LAC-4 were further studied, and the kinetics parameters were calculated using the Lineweaver-Burk double reciprocal plot method. As shown in Table 2, the Km values of LAC-4 for 2,6-DCP and 2,3,6-TCP were 3.636 mM and 4.875 mM, respectively. These results indicated that the affinity of LAC-4 for 2,6-DCP was higher than 2,3,6-TCP. In addition, the K_cat_ values for LAC-4 degradation of 2,6-DCP and 2,3,6-TCP were 1.23 s^−1^ and 0.67 s^−1^, respectively, indicating that the catalytic efficiency of LAC-4 on 2,6-DCP was also higher than 2,3,6-TCP.

The ln c-t curves for LAC-4 degradation of the two chlorophenols (the ln c-t curves of 2,6-DCP and 2,3,6-TCP degradations by LAC-4) were further plotted (Figure 6F,G), and we calculated the reaction rate constant k value. As shown in Figure 6F,G, both chlorophenol degradations by LAC-4 were first-order reactions, where the reaction rate constant for 2,6-DCP degradation by LAC-4 was 9.56 × 10^−4^ s^−1^, and the reaction rate constant for 2,3,6-TCP degradation was 1.37 × 10^−4^ s^−1^. These results indicated that the catalytic rate of LAC-4 on 2,6-DCP was significantly higher than 2,3,6-TCP when LAC-4 was used as the catalyst for the two chlorophenol reactions. The reaction rate constant results were also consistent with the 2,6-DCP and 2,3,6-TCP time-course degradation results for LAC-4.

#### 3.3.3. Degradation of Chlorophenol Mixtures by LAC-4

Degradation of the 2,6-DCP and 2,3,6-TCP mixture by LAC-4 laccase is shown in Figure 6H, indicating that LAC-4 had a good degradation effect on the chlorophenol mixture. When the concentrations of 2,6-DCP and 2,3,6-TCP in the mixture were 100 mg/L, the 12 h 2,6-DCP and 2,3,6-TCP degradation efficiency by LAC-4 reached 100% (complete degradation). When the concentrations of 2,6-DCP and 2,3,6-TCP in the mixture increased to 200 mg/L, the 12 h 2,6-DCP and 2,3,6-TCP degradation efficiencies by LAC-4 still reached 100% and 98.74%, respectively (Figure 6H). When the concentration of both chlorophenols increased to 400 mg/L, the 12 h degradation efficiencies of 2,6-DCP and 2,3,6-TCP by LAC-4 decreased, which were 82.10% and 30.52%, respectively.

#### 3.3.4. Effects of Different Concentrations of Metal Ions and Organic Solvents on the Degradation of 2,6-Dichlorophenol and 2,3,6-Trichlorophenol by LAC-4

In actual polluted environments, most chlorophenol pollutants are found in industrial waste products and industrial wastewater. In addition, actual chlorophenol pollutants usually contain large amounts of various metal salts and organic solvents at different concentrations. Thus, metal salts and organic solvents that are ubiquitous in wastewater may affect the actual chlorophenol degradation effect of laccase. Considering the above, we further studied the effects of 19 metal salts and 11 organic solvents on 2,6-DCP and 2,3,6-TCP degradation by LAC-4 to explore the tolerance of LAC-4 for different metal salts and organic solvents during degradation of the two chlorophenols.

##### Effects of Different Concentrations of Metal ions on the Degradation of 2,6-Dichlorophenol and 2,3,6-Trichlorophenol by LAC-4

2,6-DCP

**Figure 7:**
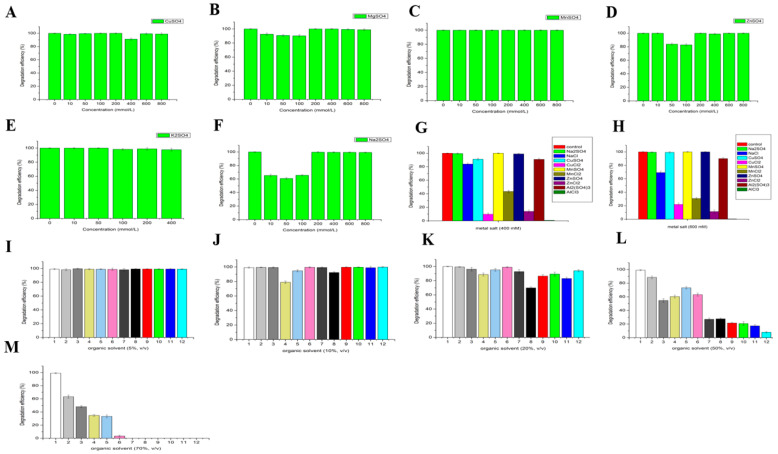
Effects of different concentrations of metal salts and organic solvents on the degradation of 2,6-DCP by LAC-4. (**A**) CuSO_4_ (**B**) MgSO_4_ (**C**) MnSO_4_ (**D**) ZnSO_4_ (**E**) K_2_SO_4_ (**F**) Na_2_SO_4_ (**G**) Comparison of the tolerance of LAC-4 for metal salts with sulfate as the anion and with chloride ions as the anion during the degradation of 2,6-DCP. The concentration of metal salt was 400 mM. Control, no addition of any metal salt. (**H**) Comparison of the tolerance of LAC-4 for metal salts with sulfate as the anion and with chloride ions as the anion during the degradation of 2,6-DCP. The concentration of metal salt was 600 mM. Control, no addition of any metal salt. (**I**) Concentration of the organic solvent was 5% (*v*/*v*). (**J**) Concentration of the organic solvent was 10% (*v*/*v*). (**K**) Concentration of the organic solvent was 20% (*v*/*v*). (**L**) Concentration of the organic solvent was 50% (*v*/*v*). (**M**) Concentration of the organic solvent was 70% (*v*/*v*). (**I**–**M**): 1: Control, no addition of any organic solvent. 2: Ethylene glycol; 3: Glycerol; 4: Butanediol; 5: Propylene glycol; 6: Methanol; 7: Ethanol; 8: DMF; 9: DMSO; 10: Acetone; 11: Isopropyl alcohol; 12: Acetonitrile.

The effects of different concentrations of metal salts on the degradation of 2,6-DCP by LAC-4 are shown in Figure 7, indicating that LAC-4 had a strong tolerance for high-concentration metal salts, such as MnSO_4_, ZnSO_4_, Na_2_SO_4_, MgSO_4_, CuSO_4_, K_2_SO_4_, during 2,6-DCP degradation. MnSO_4_, ZnSO_4_, Na_2_SO_4_, MgSO_4_, and CuSO_4_ had almost no effect on 2,6-DCP degradation by LAC-4. Even when the concentrations of MnSO_4_, ZnSO_4_, Na_2_SO_4_, MgSO_4_, CuSO_4_ in the degradation system increased to 800 mM, the 12 h 2,6-DCP degradation efficiencies by LAC-4 still reached 100%, 100%, 99.25%, 98.95%, 98.92%, respectively (Figure 7A–F).

In addition, our results indicated that with the same metal cation, the metal salt with chloride as the anion had a strong inhibitory effect on the degradation of 2,6-DCP by LAC-4, while the metal salt with sulfate as the anion had a minimal effect on the degradation of 2,6-DCP by LAC-4 (Figure 7G,H). During the degradation of 2,6-DCP, the tolerance of LAC-4 for metal salts with sulfate as the anion was considerably higher than with chloride ions as the anion. As shown in Figure 7H, when the concentrations of CuSO_4_, MnSO_4_, ZnSO_4_, Na_2_SO_4_, Al_2_(SO_4_)_3_ in the degradation system were 600 mM, the 12 h 2,6-DCP degradation efficiencies by LAC-4 reached 99.27%, 100%, 100%, 99.43%, 90.02%, respectively. However, when the concentrations of CuCl_2_, MnCl_2_, ZnCl_2_, NaCl, AlCl_3_ in the degradation system were 600 mM, the 12 h 2,6-DCP degradation efficiencies by LAC-4 decreased to 21.96%, 30.72%, 11.35%, 69.23%, 0.4%, respectively (Figure 7H). AlCl_3_ exhibited the strongest inhibitory effect on the degradation of 2,6-DCP by LAC-4, and 400 mM and 600 mM AlCl_3_ could also completely inhibit 2,6-DCP degradation by LAC-4. However, the same concentration of Al_2_(SO_4_)_3_ had almost no effect on 2,6-DCP degradation by LAC-4 (Figure 7G,H). As a result, degradation of 2,6-DCP by LAC-4 was also sensitive to metal salts, such as CdCl_2_, CoCl_2_, NiCl_2_, CaCl_2_, and when the concentration of these metal salts reached 600 mM, the 2,6-DCP degradation efficiency was less than 40%.

The effects of mixed metal salts on the degradation of 2,6-DCP by LAC-4 were further studied. The mixed metal salts (CuSO_4_ + K_2_SO_4_ + MnSO_4_ + ZnSO_4_ + MgSO_4_, where each concentration was 100 mM) did not affect 2,6-DCP degradation by LAC-4, and the degradation efficiency still reached 100%. Therefore, these results indicated that LAC-4 also had a strong tolerance for mixed metal salts during 2,6-DCP degradation.

2,3,6-TCP

The effects of different concentrations of metal salts on 2,3,6-TCP degradation by LAC-4 are shown in Figure S1. During the degradation of 2,3,6-TCP, LAC-4 also had a strong tolerance for high concentrations of metal salts, such as MnSO_4_, ZnSO_4_, Na_2_SO_4_, MgSO_4_, CuSO_4_, K_2_SO_4_ (Figure S1A–F). Similar to the degradation of 2,6-DCP, the metal salt with chloride as the anion exhibited a strong inhibitory effect on the degradation of 2,3,6-TCP by LAC-4, while the metal salt with sulfate as the anion exhibited a minimal effect on the degradation of 2,3,6-TCP by LAC-4 (Figure S1G). AlCl_3_ showed the strongest inhibitory effect on the degradation of 2,3,6-TCP by LAC-4, 200 mM AlCl_3_ could completely inhibit the degradation of 2,3,6-TCP by LAC-4.

##### Effect of Different Concentrations of Organic Solvents on the Degradation of 2,6-Dichlorophenol and 2,3,6-Trichlorophenol by LAC-4

2,6-DCP

Figure 7I–M shows the effects of 11 organic solvents at different concentrations on the degradation of 2,6-DCP by LAC-4. The degradation of 2,6-DCP by LAC-4 was not affected by the 11 organic solvents at 5% (*v*/*v*), and the degradation efficiency exceeded 98.0% (Figure 7I). The 11 organic solvents at 10% (*v*/*v*) and 20% (*v*/*v*) had a minor effect on 2,6-DCP degradation by LAC-4. For the 11 types of organic solvents at 10% (*v*/*v*), the degradation efficiency exceeded 80%. For the 11 types of organic solvents at 20% (*v*/*v*), the degradation efficiency all exceeded 70% (Figure 7J,K). Therefore, the above results indicated that for the degradation of 2,6-DCP, LAC-4 had a relatively strong tolerance for low concentrations of various organic solvents. When the organic solvent concentration further increased to 50% and 70%, the inhibitory effects of some organic solvents on the 2,6-DCP degradation by LAC-4 were substantially enhanced, and the degradation efficiency significantly declined (Figure 7L,M). However, LAC-4 showed a strong tolerance for ethylene glycol, glycerol, butanediol, propylene glycol, and methanol for the degradation of 2,6-DCP. When the concentrations of ethylene glycol, glycerol, butanediol, propylene glycol, and methanol increased to 50%, the 2,6-DCP degradation efficiencies by LAC-4 reached 88.52%, 54.64%, 60.39%, 73.28%, and 63.22%, respectively, while for the other organic solvents (50%), the degradation efficiency was less than 30% (Figure 7L). When the concentrations of ethylene glycol, glycerol, butanediol, propylene glycol, and methanol increased to 70%, the 2,6-DCP degradation efficiencies by LAC-4 still reached 63.34%, 48.16%, 34.8%, 33.39%, 3.50%, respectively. However, for the other organic solvents (70%), the degradation effect was completely lost (Figure 7M). Among the 11 organic solvents, LAC-4 had the strongest tolerance for ethylene glycol in the degradation of 2,6-DCP, and acetonitrile showed the strongest inhibitory effect on the degradation of 2,6-DCP by LAC-4. The degradation efficiency dropped sharply to 7.93% in the presence of 50% acetonitrile.

2,3,6-TCP

Figure S2 shows the effects of 11 organic solvents at different concentrations on the degradation of 2,3,6-TCP by LAC-4. The degradation of 2,3,6-TCP by LAC-4 was slightly affected by the 11 organic solvents at 5–20%, indicating that LAC-4 also had a strong tolerance for the various organic solvents at low concentrations for 2,3,6-TCP degradation (Figure S2A–C). When the concentrations of the organic solvents further increased to 50% and 70%, some of the organic solvents, such as DMSO and acetone, showed a strong inhibitory effect on 2,3,6-TCP degradation by LAC-4, and the degradation efficiency was considerably reduced. However, LAC-4 had a relatively strong tolerance for glycerol and ethylene glycol for the degradation of 2,3,6-TCP (Figure S2D,E). When the concentration increased to 50%, the degradation efficiency in the presence of ethylene glycol and glycerol could still exceed 50%. However, other organic solvents exhibited a strong inhibitory effect on degradation, such as 50% butanediol and propanediol, which caused the 2,3,6-TCP degradation efficiency by LAC-4 to be less than 20%, and 50% DMF, isopropanol, acetonitrile, and ethanol completely inhibited LAC-4 degradation of 2,3,6-TCP. When the concentrations of glycerol and ethylene glycol further increased to 70%, LAC-4 still showed a certain degradation ability on 2,3,6-TCP, and the degradation efficiencies reached 34.76% and 16.39%, respectively. However, other organic solvents at 70% completely inhibited the degradation of 2,3,6-TCP by LAC-4 (Figure S2D,E). Similar to the effect of the organic solvents on 2,6-DCP degradation by LAC-4, among the 11 organic solvents, LAC-4 had the strongest tolerance for glycerol and ethylene glycol for the degradation of 2,3,6-TCP. Specifically, DMF had the strongest inhibitory effect on the degradation of 2,3,6-TCP by LAC-4, and the degradation efficiencies reduced to 69.89% and 0% in the presence of 20% and 50% DMF, respectively.

### 3.4. Study on the Detoxification of Chlorophenols by LAC-4

The above research results indicated that LAC-4 had a good degradation effect on both 2,6-DCP and 2,3,6-TCP. Therefore, to clarify whether LAC-4 had a strong detoxification ability during chlorophenol degradation, we measured and analyzed the toxicities of the products after 2,6-DCP and 2,3,6-TCP degradation by LAC-4. We used the germination and growth of three plant seeds (mung bean, wheat, and rice) as measurement indicators, and we studied changes in the phytotoxicities of the two chlorophenols and chlorophenol mixtures at different concentrations before and after degradation by LAC-4.

#### 3.4.1. 2,6-DCP

##### Mung Bean Seed

**Figure 8:**
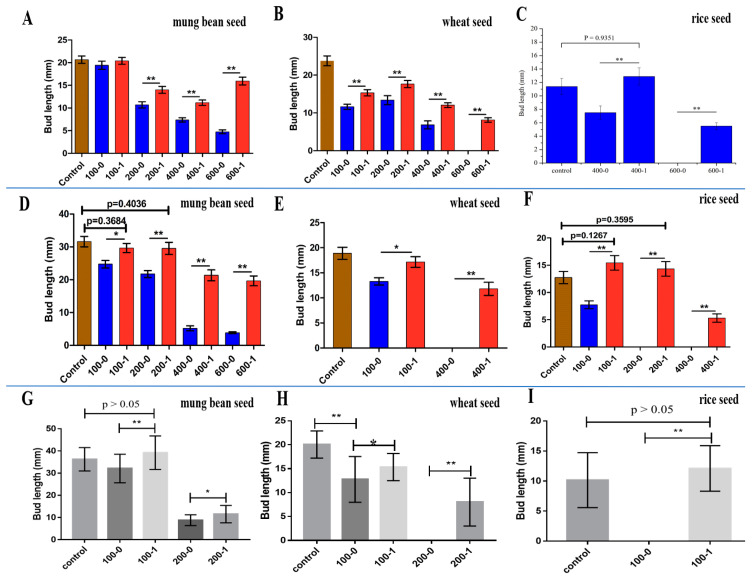
Phytotoxicities of individual chlorophenols (2,6-DCP, 2,3,6-TCP) and chlorophenol mixtures (2,6-DCP + 2,3,6-TCP) at different concentrations before and after degradation by LAC-4. The plant seed shoots in chlorophenols treated with LAC-4 laccase and the plant seed shoots exposed to untreated chlorophenols were measured, respectively. *, significant difference (*p* < 0.05); **, very significant difference (*p* < 0.01). (**A**) The toxicity measurements for the 2,6-DCP degradation products by LAC-4 on mung bean seed germination. Control: no addition of 2,6-DCP; 100-0: 100 mg/L of 2,6-DCP not treated with LAC-4; 100-1: 100 mg/L of 2,6-DCP treated with LAC-4; 200-0: 200 mg/L of 2,6-DCP not treated with LAC-4; 200-1: 200 mg/L of 2,6-DCP treated with LAC-4; 400-0: 400 mg/L of 2,6-DCP not treated with LAC-4; 400-1: 400 mg/L of 2,6-DCP treated with LAC-4; 600-0: 600 mg/L of 2,6-DCP not treated with LAC-4; 600-1: 600 mg/L of 2,6-DCP treated with LAC-4. (**B**) The toxicity measurements for the 2,6-DCP degradation products by LAC-4 on wheat seed germination. Control: no addition of 2,6-DCP; 100-0: 100 mg/L of 2,6-DCP not treated with LAC-4; 100-1: 100 mg/L of 2,6-DCP treated with LAC-4; 200-0: 200 mg/L of 2,6-DCP not treated with LAC-4; 200-1: 200 mg/L of 2,6-DCP treated with LAC-4; 400-0: 400 mg/L of 2,6-DCP not treated with LAC-4; 400-1: 400 mg/L of 2,6-DCP treated with LAC-4; 600-0: 600 mg/L of 2,6-DCP not treated with LAC-4; 600-1: 600 mg/L of 2,6-DCP treated with LAC-4. (**C**) The toxicity measurements for the 2,6-DCP degradation products by LAC-4 on rice seed germination. Control: no addition of 2,6-DCP; 400-0: 400 mg/L of 2,6-DCP not treated with LAC-4; 400-1: 400 mg/L of 2,6-DCP treated with LAC-4; 600-0: 600 mg/L of 2,6-DCP not treated with LAC-4; 600-1: 600 mg/L of 2,6-DCP treated with LAC-4. (**D**) The toxicity measurements for the 2,3,6-TCP degradation products by LAC-4 on mung bean seed germination. Control: no addition of 2,3,6-TCP; 100-0: 100 mg/L of 2,3,6-TCP not treated with LAC-4; 100-1: 100 mg/L of 2,3,6-TCP treated with LAC-4; 200-0: 200 mg/L of 2,3,6-TCP not treated with LAC-4; 200-1: 200 mg/L of 2,3,6-TCP treated with LAC-4; 400-0: 400 mg/L of 2,3,6-TCP not treated with LAC-4; 400-1: 400 mg/L of 2,3,6-TCP treated with LAC-4; 600-0: 600 mg/L of 2,3,6-TCP not treated with LAC-4; 600-1: 600 mg/L of 2,3,6-TCP treated with LAC-4. (**E**) The toxicity measurements for the 2,3,6-TCP degradation products by LAC-4 on wheat seed germination. Control: no addition of 2,3,6-TCP; 100-0: 100 mg/L of 2,3,6-TCP not treated with LAC-4; 100-1: 100 mg/L of 2,3,6-TCP treated with LAC-4; 400-0: 400 mg/L of 2,3,6-TCP not treated with LAC-4; 400-1: 400 mg/L of 2,3,6-TCP treated with LAC-4. (**F**) The toxicity measurements for the 2,3,6-TCP degradation products by LAC-4 on rice seed germination. Control: no addition of 2,3,6-TCP; 100-0: 100 mg/L of 2,3,6-TCP not treated with LAC-4; 100-1: 100 mg/L of 2,3,6-TCP treated with LAC-4; 200-0: 200 mg/L of 2,3,6-TCP not treated with LAC-4; 200-1: 200 mg/L of 2,3,6-TCP treated with LAC-4; 400-0: 400 mg/L of 2,3,6-TCP not treated with LAC-4; 400-1: 400 mg/L of 2,3,6-TCP treated with LAC-4. (**G**) The toxicity measurements for the mixed chlorophenols (2,6-DCP + 2,3,6-TCP) degradation products by LAC-4 on mung bean seed germination. Control: no addition of mixed chlorophenols; 100-0: mixed chlorophenols (100 mg/L 2,6-DCP + 100 mg/L 2,3,6-TCP) not treated with LAC-4; 100-1: mixed chlorophenols (100 mg/L 2,6-DCP + 100 mg/L 2,3,6-TCP) treated with LAC-4; 200-0: mixed chlorophenols (200 mg/L 2,6-DCP + 200 mg/L 2,3,6-TCP) not treated with LAC-4; 200-1: mixed chlorophenols (200 mg/L 2,6-DCP + 200 mg/L 2,3,6-TCP) treated with LAC-4. (**H**) The toxicity measurements for the mixed chlorophenols (2,6-DCP + 2,3,6-TCP) degradation products by LAC-4 on wheat seed germination. (**I**) The toxicity measurements for the mixed chlorophenols (2,6-DCP + 2,3,6-TCP) degradation products by LAC-4 on rice seed germination.

Figure 8A shows the toxicity measurements of the LAC-4 2,6-DCP degradation products on mung bean seed germination. Compared to the blank control group (control, with no addition of 2,6-DCP), when the 2,6-DCP concentration increased to more than 200 mg/L, the growth of mung bean shoots was noticeably inhibited, and the lengths of the shoots gradually decreased with increasing concentration of 2,6-DCP. The mung bean shoot lengths in 200 mg/L, 400 mg/L, and 600 mg/L 2,6-DCP were 51.81%, 35.69%, and 23% of the blank control group, respectively. The above results showed that when the concentration of 2,6-DCP exceeded 200 mg/L, the mung bean shoot growth was significantly inhibited, and the higher the concentration of 2,6-DCP, the stronger the inhibitory effect. Thus, undegraded 2,6-DCP showed toxicity on the germination and growth of mung bean seeds.

As shown in Figure 8A, the mung bean seed shoots in 2,6-DCP (200, 400, and 600 mg/L) treated with LAC-4 laccase were significantly longer than the mung bean shoots exposed to untreated 2,6-DCP (200, 400, and 600 mg/L), with extremely significant differences (*p* < 0.01). The mung bean seed shoots in 2,6-DCP (200, 400, and 600 mg/L) treated with LAC-4 laccase were 1.31, 1.52, and 3.36 times longer than the mung bean shoots in untreated 2,6-DCP (200, 400, and 600 mg/L), respectively (Figure 8A). Therefore, the above results showed that compared to the undegraded 2,6-DCP, the products from 2,6-DCP degradation by LAC-4 were considerably less toxic to the germination of mung bean seeds. Furthermore, the inhibitory effects of the degradation products on the germination and growth of mung bean seeds were greatly reduced. As a result, LAC-4 had a good detoxification ability on the toxicity of 2,6-DCP on mung bean seeds. Among the three concentrations of 2,6-DCP, LAC-4 had the strongest detoxification ability on the mung bean seeds in 600 mg/L 2,6-DCP.

##### Wheat Seed

Toxicity measurements of the 2,6-DCP products from LAC-4 laccase treatment on wheat seed germination are shown in Figure 8B. Compared to the blank control group (without 2,6-DCP), the undegraded 2,6-DCP significantly affected the germination and growth of the wheat seeds. The lengths of the wheat shoots in 100 mg/L, 200 mg/L, and 400 mg/L 2,6-DCP were 48.89%, 56.24%, and 28.71% of the blank control, respectively, and 600 mg/L 2,6-DCP completely inhibited wheat seed germination. The above results indicated that undegraded 2,6-DCP (above 100 mg/L) showed significant toxicity on the germination and growth of wheat seeds, and 2,6-DCP at 600 mg/L was the most toxic to the germination and growth of wheat seeds.

As shown in Figure 8B, the wheat seed shoots in 2,6-DCP (100, 200, 400, and 600 mg/L) treated with LAC-4 laccase were significantly longer than the wheat seed shoots in untreated 2,6-DCP (100, 200, 400, and 600 mg/L), with extremely significant differences (*p* < 0.01). The wheat seed shoots in 2,6-DCP (100, 200, and 400 mg/L) treated with LAC-4 laccase were 1,32, 1.32, and 1.76 times longer than the wheat seed shoots in untreated 2,6-DCP (100, 200, and 400 mg/L), respectively. Furthermore, untreated 600 mg/L 2,6-DCP would completely inhibit the wheat seed germination, while 600 mg/L 2,6-DCP treated with LAC-4 exhibited a significantly weakened toxic effect on wheat seed germination. The germination of wheat seeds in 600 mg/L 2,6-DCP treated with LAC-4 could be also restored to a certain extent (Figure 8B). Thus, the above results indicated that the products of 2,6-DCP degradation by LAC-4 were also significantly less toxic to wheat seed germination, and the inhibitory effects of the degradation products on wheat seed germination and growth were considerably reduced.

We also studied the effects of different concentrations of 2,6-DCP on the germination rates of wheat seeds before and after degradation. As shown in Table 3, as the concentration of 2,6-DCP increased, the germination rates of the wheat seeds considerably declined. When the concentrations of 2,6-DCP were 200 and 400 mg/L, the germination rates were 80% and 60%, respectively. However, when the concentration of 2,6-DCP increased to 600 mg/L, wheat seed germination did not occur. The germination rates of the wheat seeds in 2,6-DCP (200, 400, 600 mg/L) treated with LAC-4 laccase were significantly higher than the wheat seeds in untreated 2,6-DCP (200, 400, 600 mg/L). The germination rates of the seeds in 200 mg/L and 400 mg/L 2,6-DCP treated by LAC-4 were restored to normal (95% and 100%), and the germination rate of the seeds in 600 mg/L 2,6-DCP after LAC-4 treatment also increased to 80%. The germination rate of the wheat seeds in 600 mg/L 2,6-DCP without LAC-4 treatment was 0, while the germination rate of the wheat seeds in 600 mg/L 2,6-DCP treated with LAC-4 increased to 80%, indicating that LAC-4 treatment greatly reduced the toxicity of 600 mg/L 2,6-DCP on wheat seed germination.

Based on the above results, untreated 2,6-DCP had serious inhibitory and toxic effects on the growth and germination rates of wheat seeds, while the inhibitory effects and toxicity of 2,6-DCP treated with LAC-4 on wheat seed growth and germination were substantially weakened. Thus, LAC-4 also had a strong detoxification effect on the toxicity of 2,6-DCP on wheat seeds.

##### Rice Seed

The toxicity measurements for the 2,6-DCP degradation products by LAC-4 on rice seed germination are shown in Figure 8C. The rice seed shoots in 2,6-DCP (400, 600 mg/L) treated with LAC-4 laccase were significantly longer than the rice seed shoots in untreated 2,6-DCP (400, 600 mg/L), with extremely significant differences (*p* < 0.01). The rice seed shoots in 2,6-DCP (400 mg/L) treated with LAC-4 were 1.72 times longer than the rice seeds in untreated 2,6-DCP (400 mg/L). In addition, we found that the shoot lengths of the rice seeds in 2,6-DCP (400 mg/L) treated with LAC-4 were not significantly different from the blank control group (*p* > 0.05). The results indicated that LAC-4 treatment could completely eliminate the toxic effects of 2,6-DCP (400 mg/L) on the germination of rice seeds (Figure 8C). Untreated 600 mg/L 2,6-DCP could completely inhibit the rice seed germination, while 600 mg/L 2,6-DCP treated with LAC-4 showed a significantly weakened toxic effect on rice seed germination. The germination of rice seeds in 600 mg/L 2,6-DCP treated with LAC-4 could be restored to a certain extent. Thus, LAC-4 treatment of 2,6-DCP could completely eliminate (400 mg/L 2,6-DCP) or weaken (600 mg/L 2,6-DCP) the toxic effects on rice seeds.

We also studied the effects of different concentrations of 2,6-DCP on the germination rates of rice seeds before and after degradation. As shown in Table 4, when the 2,6-DCP concentration was 400 mg/L, the germination rate reduced to 70%, while 600 mg/L of 2,6-DCP would completely inhibit rice seed germination (germination rate was 0). However, the germination rates of the rice seeds in 2,6-DCP (400, 600 mg/L) treated with LAC-4 laccase were considerably higher compared to untreated 2,6-DCP (400, 600 mg/L). The germination rates after exposure to 400 mg/L and 600 mg/L of 2,6-DCP treated by LAC-4 increased to 85% and 50%, respectively. The germination rate of the rice seeds in 600 mg/L 2,6-DCP without LAC-4 treatment was 0, while the germination rate of the rice seeds in 600 mg/L 2,6-DCP treated with LAC-4 increased to 50%, indicating that LAC-4 treatment significantly reduced the toxicity of 600 mg/L 2,6-DCP on rice seed germination.

The above results indicated that the products resulting from 2,6-DCP degradation by LAC-4 on rice seed germination were considerably less toxic, and treatment by LAC-4 could completely eliminate the toxicity of 400 mg/L 2,6-DCP on rice seeds. Thus, LAC-4 laccase had a strong detoxification effect on the toxicity of 2,6-DCP on rice seeds.

#### 3.4.2. 2,3,6-TCP

##### Mung Bean Seed

Toxicity measurements of the 2,3,6-TCP products from LAC-4 laccase treatment on mung bean seed germination are shown in Figure 8D. The higher the concentration of 2,3,6-TCP, the stronger the inhibitory effect on mung bean seed shoot growth, and the shoot lengths of the mung bean seeds in 100 mg/L, 200 mg/L, 400 mg/L, and 600 mg/L of 2,3,6-TCP were 78%, 69%, 16%, and 12% of the blank control group, respectively. The above results indicated that the undegraded 2,3,6-TCP was especially toxic to the germination and growth of mung bean seeds.

The mung bean seed shoots in 2,3,6-TCP (100, 200, 400, and 600 mg/L) treated with LAC-4 laccase were significantly longer than the mung bean shoots exposed to untreated 2,3,6-TCP (100, 200, 400, and 600 mg/L) (*p* < 0.01). The shoot of the mung bean seeds in 2,3,6-TCP (100, 200, 400, and 600 mg/L) treated with LAC-4 laccase were 1.20, 1.36, 4.10 and 5.09 times longer than the mung bean seeds in untreated 2,3,6-TCP (100, 200, 400, and 600 mg/L), respectively. There were no significant differences between the shoot lengths of the mung bean seeds in 2,3,6-TCP (100, 200 mg/L) treated with LAC-4 and the blank control (without 2,3,6-TCP, control) (*p* > 0.05) (Figure 8D). Thus, the results indicated that LAC-4 laccase treatment could completely remove the toxic effect of 2,3,6-TCP (100, 200 mg/L) on mung bean seeds. For higher concentrations of 2,3,6-TCP (400, 600 mg/L), LAC-4 treatment could effectively reduce 2,3,6-TCP toxicity on mung bean seeds.

The above results indicated substantially reduced toxicity of the 2,3,6-TCP degradation products by LAC-4 on the germination of mung bean seeds, and LAC-4 laccase treatment could completely eliminate the toxicity of 100 and 200 mg/L 2,3,6-TCP on the mung bean seeds. Thus, LAC-4 laccase had a good detoxification effect on the toxicity of 2,3,6-TCP on mung bean seeds.

##### Wheat Seed

Toxicity measurements of the 2,3,6-TCP products from LAC-4 laccase treatment on wheat seed germination are shown in Figure 8E. Compared to the blank control group, 100 mg/L and 400 mg/L 2,3,6-TCP significantly inhibited wheat seed germination, and 400 mg/L 2,3,6-TCP completely inhibited wheat seed germination. Thus, the above results demonstrated that undegraded 2,3,6-TCP was highly toxic to the germination and growth of wheat seeds.

The wheat seed shoots in 2,3,6-TCP (100 and 400 mg/L) treated with LAC-4 laccase were significantly longer than the wheat shoots exposed to untreated 2,3,6-TCP (100 and 400 mg/L) (*p* < 0.01). The shoots of the wheat seeds in 2,3,6-TCP (100 mg/L) after LAC-4 laccase treatment were 1.29 times longer than the wheat seeds in untreated 2,3,6-TCP (100 mg/L), and the average value of the shoot length of wheat seeds in 400 mg/L 2,3,6-TCP after LAC-4 treatment was 11 mm (Figure 8E).

The effects of different 2,3,6-TCP concentrations on the germination rates of wheat seeds before and after degradation are shown in Table 5. When the 2,3,6-TCP concentration was 100 mg/L, the germination rate of the wheat seeds was only 35%, while 400 mg/L 2,3,6-TCP completely inhibited wheat seed germination (the germination rate was 0). However, the germination rates of the wheat seeds in 100 mg/L and 400 mg/L 2,3,6-TCP after LAC-4 treatment reached 95% and 25%, respectively, and the germination rate was significantly higher than undegraded 2,3,6-TCP. After LAC-4 treatment, the germination rate of the wheat seeds in 100 mg/L 2,3,6-TCP was restored to normal levels.

The above results indicated that LAC-4 treatment could considerably reduce 2,3,6-TCP toxicity on wheat seed germination, and LAC-4 laccase also had a good detoxification effect for 2,3,6-TCP toxicity on wheat seeds.

##### Rice Seed

As shown in Figure 8F, undegraded 2,3,6-TCP was more toxic to rice seed germination, and even 200 mg/L 2,3,6-TCP completely inhibited rice seed germination. The rice seed shoots in 2,3,6-TCP (100, 200, 400 mg/L) treated with LAC-4 laccase were significantly longer than the rice seed shoots in untreated 2,3,6-TCP (100, 200, 400 mg/L) (*p* < 0.01). The shoots of the rice seeds in 2,3,6-TCP (100 mg/L) treated with LAC-4 laccase were 2.00 times longer than the rice seeds in untreated 2,3,6-TCP (100 mg/L) rice seeds. The shoot lengths of the rice seeds in 200 mg/L and 400 mg/L 2,3,6-TCP after LAC-4 treatment were 14 mm and 6 mm (average value), respectively, while the shoot lengths of the rice seeds in untreated 2,3,6-TCP were zero (Figure 8F).

The shoot lengths of the rice seeds in 2,3,6-TCP (100, 200 mg/L) treated with LAC-4 did not significantly differ from the blank control group (without 2,3,6-TCP) (*p* > 0.05). Therefore, these results showed that treatment with LAC-4 laccase could completely eliminate the toxicity of 2,3,6-TCP (100, 200 mg/L) on the rice seeds (Figure 8F).

The effects of different 2,3,6-TCP concentrations on the germination rates of rice seeds before and after degradation are shown in Table 5. The germination rates of the rice seeds in untreated 100, 200, and 400 mg/L 2,3,6-TCP were 75%, 0%, and 0%, respectively, and 200 and 400 mg/L 2,3,6-TCP completely inhibited rice seed germination. The germination rates of the rice seeds in 100, 200, and 400 mg/L 2,3,6-TCP treated with LAC-4 increased to 80%, 80%, and 50%, respectively, and the effect of LAC-4 laccase treatment on the promotion of rice seed germination in 200 and 400 mg/L 2,3,6-TCP was particularly significant. The germination rates of the rice seeds in 2,3,6-TCP treated with LAC-4 greatly improved.

The above results indicated that treatment with LAC-4 could completely remove or weaken the toxicity of 2,3,6-TCP on rice seeds, and LAC-4 also had a strong detoxification effect against the toxicity of 2,3,6-TCP on rice seeds.

#### 3.4.3. 2,6-DCP + 2,3,6-TCP

The above research results indicated that LAC-4 laccase possessed strong detoxification ability for the phytotoxicity of individual chlorophenol. LAC-4 also had a good degradation effect on chlorophenol mixtures (2,6-DCP + 2,3,6-TCP); therefore, we further studied the detoxification effect of LAC-4 on the phytotoxicities of mixed chlorophenols (2,6-DCP + 2,3,6-TCP).

##### Mung Bean Seeds

The degradation product toxicity measurements for the mixed chlorophenols (2,6-DCP + 2,3,6-TCP) treated with LAC-4 laccase on mung bean seed germination are shown in Figure 8G. The mung bean seed shoots in the chlorophenol mixture treated with LAC-4 laccase (100 mg/L + 100 mg/L, 200 mg/L + 200 mg/L) were significantly longer than the mung bean shoots in the untreated chlorophenol mixture (100 mg/L + 100 mg/L, 200 mg/L + 200 mg/L). The mung bean shoots in the chlorophenol mixture (2,6-dichlorophenol + 2,3,6-trichlorophenol, 100 mg/L + 100 mg/L) treated with LAC-4 laccase were 1.22 times longer than the mung bean seed shoots in the untreated chlorophenol mixture (2,6-dichlorophenol + 2,3,6-trichlorophenol, 100 mg/L + 100 mg/L). The mung bean shoots in the chlorophenol mixture (2,6-dichlorophenol + 2,3,6-trichlorophenol, 200 mg/L + 200 mg/L) treated with LAC-4 laccase were 1.31 times longer than the mung bean seed shoots in the untreated chlorophenol mixture (2,6-dichlorophenol + 2,3,6-trichlorophenol, 200 mg/L + 200 mg/L) (Figure 8G).

In addition, there were no significant differences between the shoot lengths of the mung bean seeds in the chlorophenol mixture treated with LAC-4 (2,6-dichlorophenol + 2,3,6-trichlorophenol, 100 mg/L + 100 mg/L) and the blank control (without the chlorophenol mixture) (*p* > 0.05) (Figure 8G). Thus, the results indicated that LAC-4 laccase treatment could completely remove the toxic effect of the chlorophenol mixture (2,6-dichlorophenol + 2,3,6-trichlorophenol, 100 mg/L + 100 mg/L) on mung bean seeds.

The effects of different concentrations of mixed chlorophenol on the germination rates of mung bean seeds before and after degradation are shown in Table 6. The germination rate of the mung bean seeds in the chlorophenol mixture (2,6-dichlorophenol + 2,3,6-trichlorophenol, 200 mg/L + 200 mg/L) treated with LAC-4 laccase could be restored to 100%.

The above results indicated that treatment with LAC-4 could completely remove or weaken the toxicity of the chlorophenol mixture on mung bean seeds, and LAC-4 had a good detoxification effect on the toxicity of the chlorophenol mixture (2,6-dichlorophenol + 2,3,6-trichlorophenol) on mung bean seeds.

##### Wheat Seeds

The degradation product toxicity measurements for the mixed chlorophenols treated with LAC-4 laccase on wheat seed germination are shown in Figure 8H. Mixed chlorophenols significantly inhibited wheat seed germination. In addition, the chlorophenol mixture (2,6-dichlorophenol + 2,3,6-trichlorophenol, 200 mg/L + 200 mg/L) completely inhibited wheat seed germination, indicating that the mixed chlorophenols were highly toxic to wheat seed germination. The shoots of the wheat seeds in the chlorophenol mixture (2,6-dichlorophenol + 2,3,6-trichlorophenol, 100 mg/L + 100 mg/L) treated with LAC-4 laccase were 1.20 times longer than the wheat seeds in the untreated chlorophenol mixture (2,6-dichlorophenol + 2,3,6-trichlorophenol, 100 mg/L + 100 mg/L), and the shoot lengths of the wheat seeds in higher concentrations of chlorophenol mixture (2,6-dichlorophenol + 2,3,6-trichlorophenol, 200 mg/L + 200 mg/L) treated with LAC-4 laccase could be restored to a certain extent (Figure 8H).

The effects of different concentrations of mixed chlorophenol on the germination rates of wheat seeds before and after degradation are shown in Table 6. Compared to the untreated chlorophenol mixture, the wheat seed germination rate of the chlorophenol mixture treated with LAC-4 laccase considerably improved, and the germination rate of the wheat seeds in the 100 mg/L mixed chlorophenol solution treated with LAC-4 was essentially restored to normal (the germination rate reached 90%).

The above results indicated that LAC-4 laccase treatment could effectively reduce chlorophenol mixture toxicity on wheat seeds. LAC-4 also had a good detoxification effect on the toxicity of mixed chlorophenols on wheat seeds.

##### Rice Seeds

The toxicity measurements for the mixed chlorophenol degradation products after LAC-4 laccase treatment on rice seed germination are shown in Figure 8I. We observed that the untreated chlorophenol mixture was extremely toxic to the germination of rice seeds, and the 2,6-DCP + 2,3,6-TCP mixture (100 mg/L + 100 mg/L) completely inhibited rice seed germination. However, there were no significant differences in the shoot lengths between the rice seeds in the chlorophenol mixture (2,6-dichlorophenol + 2,3,6-trichlorophenol, 100 mg/L + 100 mg/L) treated with LAC-4 laccase and the blank control (without chlorophenol mixture addition) (*p* > 0.05) (Figure 8I). These results showed that treatment with LAC-4 laccase could completely eliminate chlorophenol mixture (2,6-dichlorophenol + 2,3,6-trichlorophenol, 100 mg/L + 100 mg/L) toxicity on rice seeds. In addition, the germination rate results also showed that the germination rate of the rice seeds in the chlorophenol mixture (2,6-dichlorophenol + 2,3,6-trichlorophenol, 100 mg/L + 100 mg/L) treated with LAC-4 laccase was restored to 100%, while the germination rate of the rice seeds in the chlorophenol mixture without LAC-4 treatment was 0. Therefore, the above results indicated that LAC-4 laccase also had strong detoxification ability for mixed chlorophenol toxicity on rice seeds.

In summary, untreated individual chlorophenols (2,6-DCP or 2,3,6-TCP) and the chlorophenol mixture (2,6-DCP + 2,3,6-TCP) were both highly toxic to the germination and growth of three plant seeds, and treatment with LAC-4 laccase significantly reduced or completely eliminated individual chlorophenol and chlorophenol mixture toxicities on the three types of plant seeds. Thus, LAC-4 treatment had a strong detoxification ability and a good detoxification effect on the phytotoxicity of individual chlorophenols and chlorophenol mixtures, and the phytotoxicities of 2,6-DCP and 2,3,6-TCP treated with LAC-4 were considerably reduced or eliminated.

### 3.5. Study on the LAC-4 Degradation Mechanism of Chlorophenols

The above research results showed that LAC-4 laccase had strong degradation and good detoxification effects on 2,6-DCP and 2,3,6-TCP. Based on the above results, we further explored the degradation mechanisms and pathways of the two chlorophenols by LAC-4, and we identified and analyzed the metabolites of 2,6-DCP and 2,3,6-TCP conversion by LAC-4.

#### 3.5.1. 2,6-DCP

##### Study of the Dechlorination of 2,6-Dichlorophenol and 2,3,6-Trichlorophenol by LAC-4

Previous studies have shown that laccase-mediated free radical coupling or nucleophilic attack could lead to the dechlorination of chlorophenols [34,35]. In this study, we added silver nitrate to different degradation samples to confirm the dechlorination reaction of LAC-4 in the process of degrading 2,6-dichlorophenol and 2,3,6-trichlorophenol. As shown in Figure S3A, when free chloride ions were present in the samples, they could combine with silver ions to form a silver chloride precipitate, resulting in a cloudy solution. Ultra-pure water, LAC-4 laccase solution, 2,6-DCP solution and 2,3,6-TCP solution did not show turbidity, while the NaCl solution (3), LAC-4-degraded 600 mg/L 2,6-DCP product (5) and LAC-4-degraded 400 mg/L 2,3,6-TCP product (7) changed from clear to turbid solutions, indicating the release of chloride ions. However, the turbidity of (5) and (7) was significantly lower than that of (3), and the solution of (7) was not as clear as that of (5) (Figure S3A). The above results indicate that during the degradation of 2,6-DCP and 2,3,6-TCP by LAC-4, only a few molecules broke the C-Cl bond to release chloride ions.

Silver nitrate turbidimetry can detect the presence of trace chloride ions, and the turbidimetric method is used to quantitatively determine the dechlorination of 2,6-DCP and 2,3,6-TCP by LAC-4. The standard curve of chloride ion concentration is shown in Figure S3B. Through calculation, the amount of free chloride ions released by LAC-4-degraded 600 mg/L 2,6-DCP and 400 mg/L 2,3,6-TCP was 6.852 μg/mL and 1.374 μg/mL, respectively. It is assumed that each molecule participating in the reaction dechlorinates only one chlorine atom, i.e., during the degradation process, 5.24% of 2,6-DCP was dechlorinated and 1.91% of 2,3,6-TCP was dechlorinated.

##### Identification of Intermediate Products of 2,6-Dichlorophenol Degraded by LAC-4 and Investigation of Degradation Pathways

**Figure 9:**
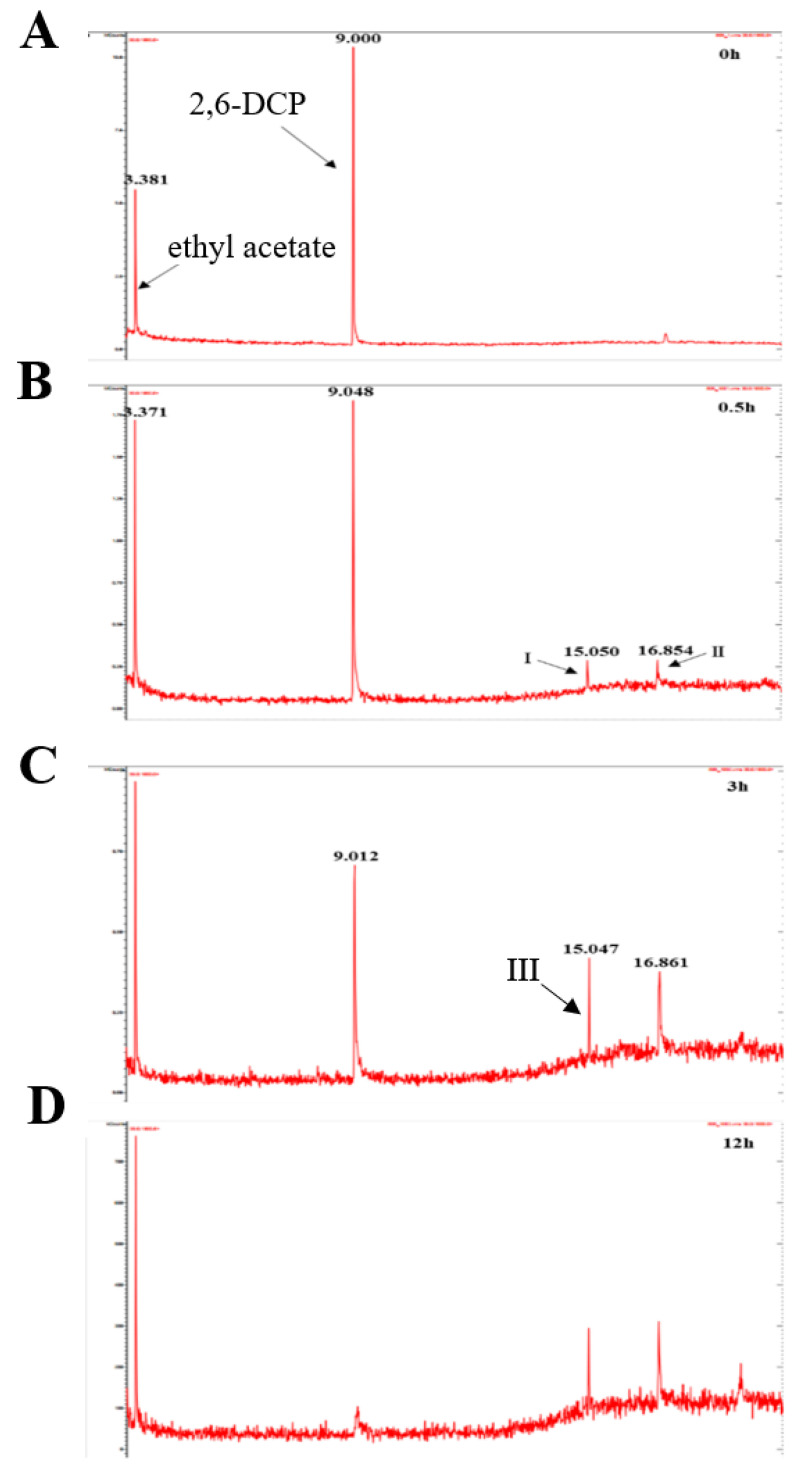
Gas chromatography (GC) peak spectra of degradation products of 2,6-DCP degraded by LAC-4 at different time points. (**A**) 0 h, (**B**) 0.5 h, (**C**) 3 h, (**D**) 12 h.

**Figure 10:**
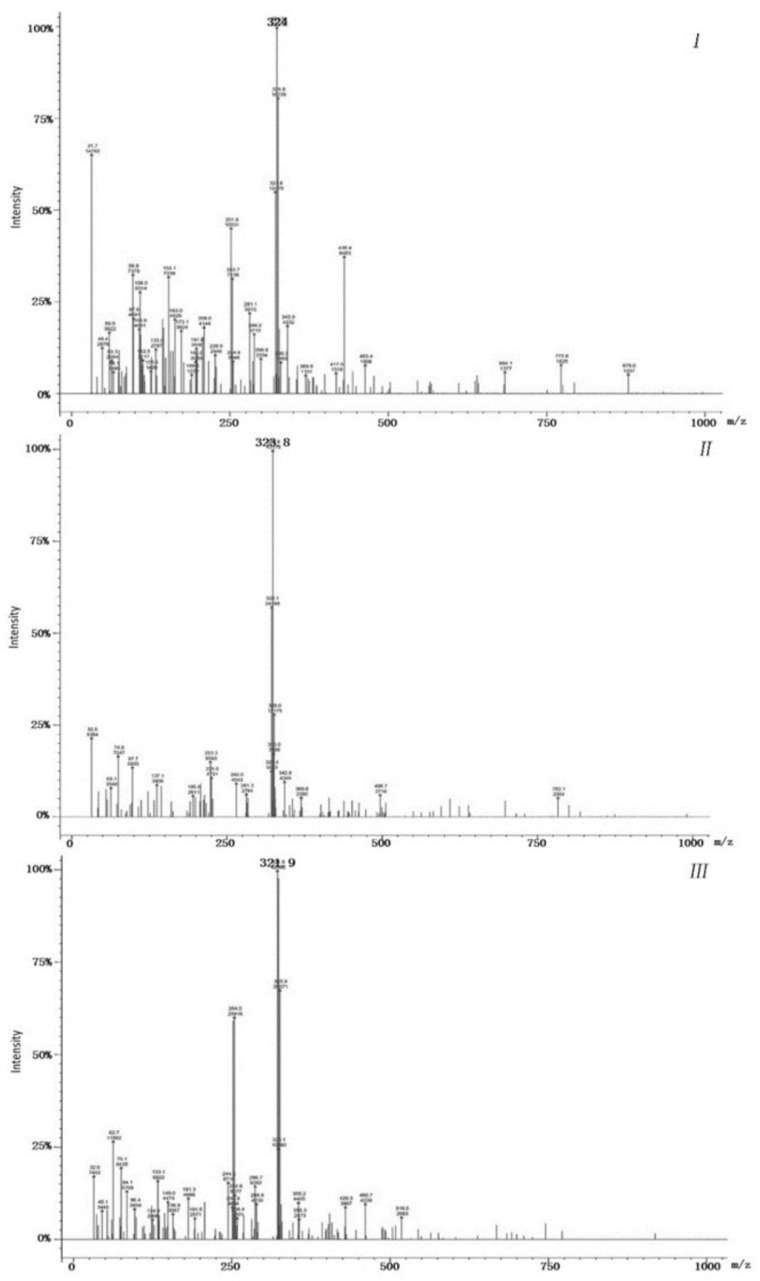
Mass spectrum (MS) of degradation products of 2,6-DCP degraded by LAC-4. Three products were detected by GC-MS analysis: the m/z values of substances I, II and III were 324, 323.8 and 321.9, respectively.

GC-MS analysis was performed on the degradation samples at 0, 0.5, 3 and 12 h of LAC-4-degraded 2,6-DCP to identify the intermediate degradation products. The results of GC-MS are shown in Figure 9 and Figure 10. In the 0 h degraded sample, only two substances, ethyl acetate and 2,6-DCP, were detected by MS. In the 0.5 h degraded sample, two new peaks (I and II) appeared in addition to two substances at 0 h, 15.050 and 16.854 min, *m*/*z* were 324 and 323.8, respectively (Figure 9). The 3 h degraded sample still showed four substance peaks, and the retention time was generally the same as that of the 0.5 h sample. The substance *m*/*z* with retention time 16.861 min was 324, indicating that the substance was still I, but the *m*/*z* of peak (III) with retention time 15.047 min was different from that of I, which was 321.9. Therefore, three products were detected by GC-MS analysis: the *m*/*z* values of substances I, II and III were 324, 323.8 and 321.9, respectively (Figure 10). The *m*/*z* data suggested that LAC-4 catalyzed the polymerization of 2,6-DCP to form dimers.

**Figure 11:**
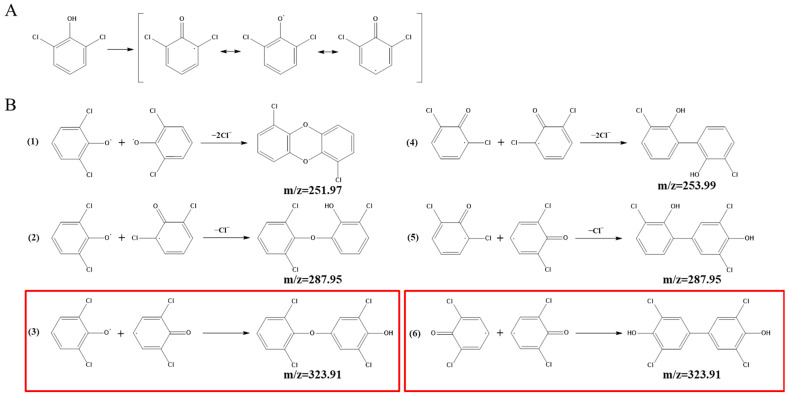
Free radicals generated by 2,6-DCP catalyzed by LAC-4 laccase (**A**) and the coupling reaction between the free radicals (**B**). 2,6-DCP was first oxidized by LAC-4 laccase, generating three chemically stable free radicals (**A**); then, six possible coupling reactions randomly occurred between the free radical monomers (**B**).

Studies have shown that laccase catalyzes the formation of free radicals by chlorophenols, and an oxidative coupling reaction occurs between the free radicals. The binding positions of the two free radical monomers in the coupling reaction are related to the position of the lone pair electrons of the free radicals [30]. Based on the above GC-MC results, we speculate that 2,6-DCP was first oxidized by LAC-4 laccase to transfer one electron and one proton, generating three chemically stable free radicals (cationic radicals formed by meta-attack, unstable); then, six possible coupling reactions randomly occurred between the free radical monomers (Figure 11). Among them, chloride ions were released during the reactions (2), (4) and (5). Due to the higher activity of free radicals in the form of oxygen and para positions, lone pair electrons were more likely to react with them. The O-para, O-O and para-para couplings took precedence over other couplings. The O-O reaction involves the ortho-chlorine atom, so reaction (1) in Figure 11 was less likely to occur. Therefore, in conjunction with the *m*/*z* values of several products, it is postulated that substances I and II detected by GC-MS should be the dimers produced by reactions (3) and (6), respectively: 2,6-dichloro-4-(2,6-dichlorophenoxy) phenol and 3,3′,5,5′-tetrachloro-4,4′-dihydroxybiphenyl. In this work, the dechlorination of 2,6-DCP showed that a small amount of chloride ion was released, which confirmed that the reactions (2), (4) and (5) occurred; however, the reaction products may not be detected by GC-MS due to limited amounts. In general, LAC-4 laccase catalyzes 2,6-DCP to generate free radicals, and the free radicals further undergo oxidative coupling; the products formed are shown in Figure 11B. The free radicals generated in the process of LAC-4-catalyzed 2,6-dichlorophenol degradation and the coupling reaction between the free radicals are shown in Figure 11A,B.

**Figure 12:**
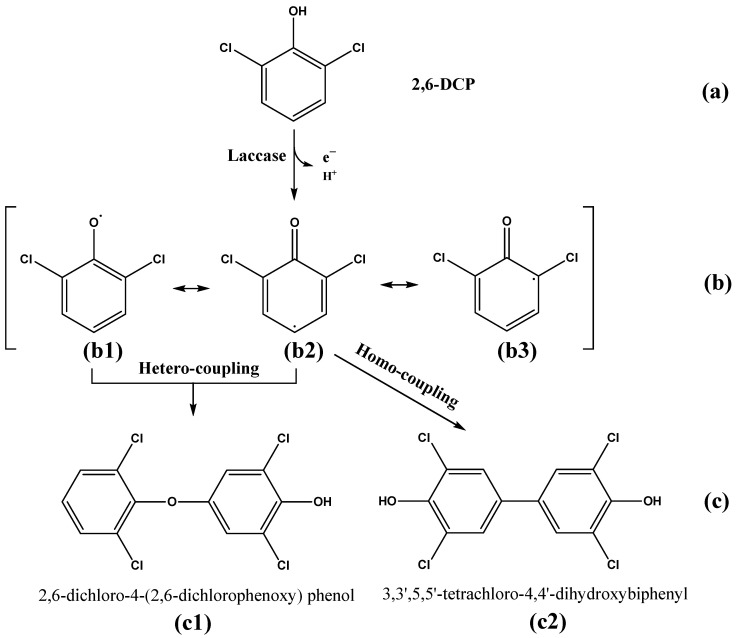
Speculation of the degradation pathway of 2,6-DCP catalyzed by LAC-4 laccase. The putative degradation pathway of 2,6-DCP catalyzed by LAC-4 laccase. (**a**) 2,6-DCP; (**b**) free radicals; (**c**) dimer products.

Based on the above analytical results, we speculate that the specific pathway through which LAC-4 catalyzes the conversion of 2,6-dichlorophenol is as follows (Figure 12): In the process of LAC-4 catalysis of 2,6-dichlorophenol, 2,6-DCP first undergoes LAC-4 oxidation and transfers one electron and one proton to generate three chemically stable free radicals, and then six possible coupling reactions occur randomly between free radical monomers. Being influenced by various factors, LAC-4 catalyzes 2,6-dichlorophenol into two main substances: 2,6-dichloro-4-(2,6-dichlorophenoxy) phenol and 3,3′,5,5′-tetrachloro-4,4′-dihydroxybiphenyl. In short, the free radicals formed by LAC-4 oxidation of 2,6-dichlorophenol produced dimers through polymerization. The putative degradation pathway of LAC-4 to 2,6-DCP is shown in Figure 12.

In summary, GC-MS identified the degradation of 2,6-DCP by LAC-4 to produce two products with *m*/*z* of 324 and 323.8, and it is speculated that these two products are 2,6-dichloro-4-(2,6-dichlorophenoxy) phenol and 3,3′,5,5′-tetrachloro-4,4′-dihydroxybiphenyl, respectively.

#### 3.5.2. 2,3,6-TCP

##### Identification of Intermediate Products of 2,3,6-Trichlorophenol Degraded by LAC-4 and Investigation of Degradation Pathways

**Figure 13:**
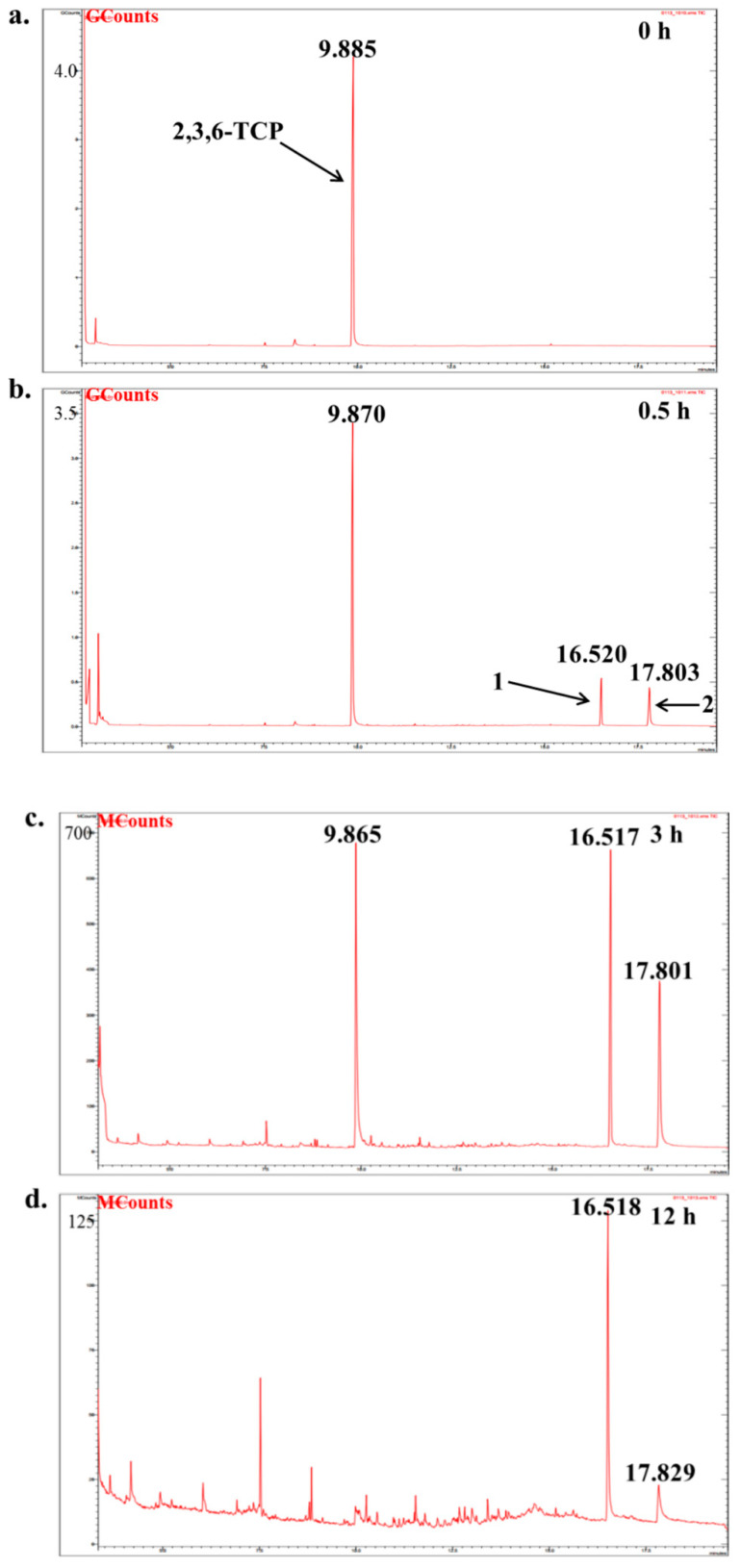
Gas chromatography (GC) peak spectra of degradation products of 2,3,6-TCP degraded by LAC-4 at different time points. (**a**) 0 h, (**b**) 0.5 h, (**c**) 3 h, (**d**) 12 h.

The intermediate products resulting from 2,3,6-TCP degradation by LAC-4 were analyzed by gas chromatography-mass spectrometry (GC-MS), and the GC detection results of 2,3,6-TCP degradation by LAC-4 at different points in time are shown in Figure 13. At the beginning of the reaction (0 h), only one substance peak was detected, which was 2,3,6-TCP (9.885 min). After reacting for 0.5 h, two new substance peaks appeared (substance 1 (16.517 min) and substance 2 (17.801 min)). With prolonged reaction time, the 2,3,6-TCP peak gradually decreased, and the peaks for the new substances gradually increased. After 12 h, the peak for 2,3,6-TCP almost completely disappeared (Figure 13).

**Figure 14:**
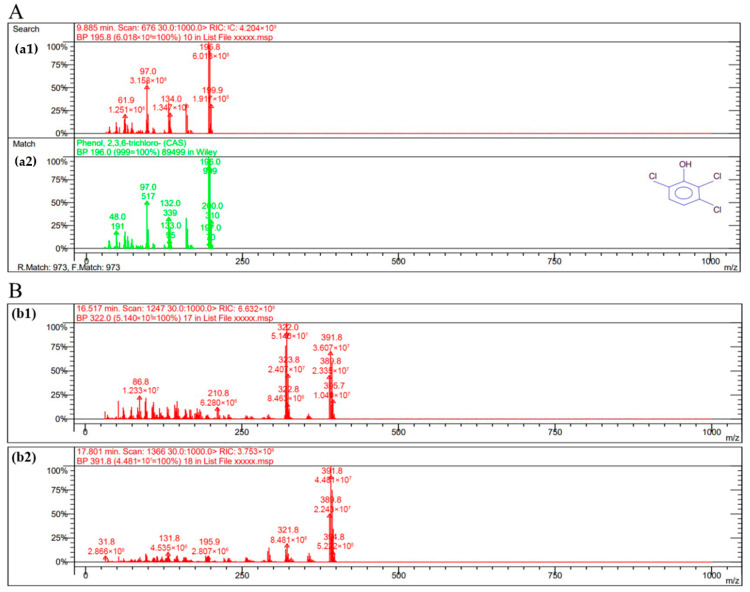
Mass spectrum (MS) of degradation products of 2,3,6-TCP degraded by LAC-4. (**A**) Mass spectrum of 2,3,6-TCP. (**a1**) is the MS detection result of 2,3,6-trichlorophenol (not degraded). (**a2**) is the database matching result. (**B**) Two intermediate products were detected by GC-MS: product I (**b1**), which had a retention time of 16.517 min with a *m*/*z* of 391.8, and product II (**b2**), which had a retention time of 17.801 min with a *m*/*z* of 391.8.

The MS results of the two intermediate products produced during the conversion process of 2,3,6-TCP by LAC-4 are shown in Figure 14. GC-MS detected two intermediate products: product I, which had a retention time of 16.517 with a *m*/*z* of 391.8, and product II, which had a retention time of 17.801 with a *m*/*z* of 391.8. The *m*/*z* of 2,3,6-TCP was 196, and the *m*/*z* values of the two intermediate products from the degradation of 2,3,6-TCP by LAC-4 were identical (both were 391.8) (Figure 14). Based on the above data, we speculated that during the degradation of 2,3,6-TCP, LAC-4 completed the conversion of 2,3,6-TCP through polymerization, forming dimers. This was similar to the mechanism by which LAC-4 degraded 2,6-DCP.

**Figure 15:**
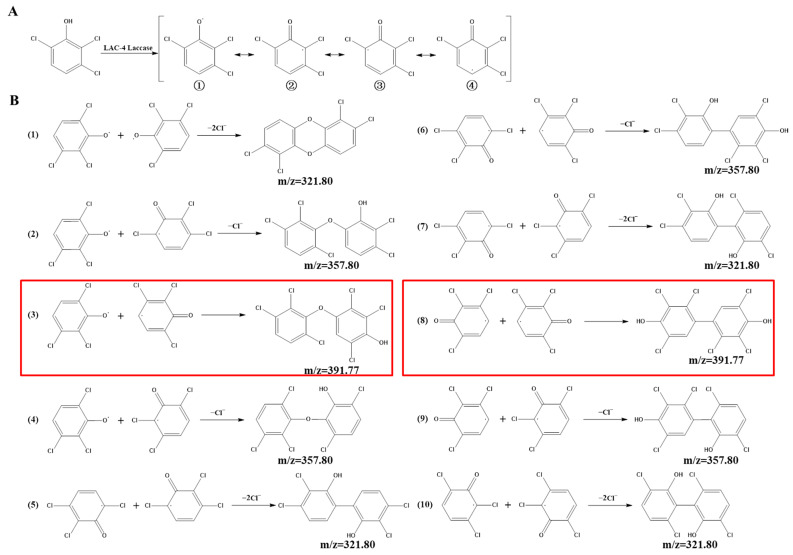
Free radicals generated by 2,3,6-TCP catalyzed by LAC-4 laccase (**A**) and the coupling reaction between the free radicals (**B**). 2,3,6-TCP was first oxidized by LAC-4 laccase, generating four chemically stable free radicals (**A**, ①–④); then, ten possible coupling reactions randomly occurred between the free radical monomers (**B**, (1)–(10)).

Based on the above results, we speculated that the degradation pathway for the catalytic conversion of 2,3,6-TCP by LAC-4 was as follows. First, under the catalytic action of LAC-4 laccase, 2,3,6-TCP lost an electron, forming four different free radicals (Figure 15A). The free radicals underwent further coupling reactions, and random coupling among the four free radicals produced ten possible products (Figure 15B). Eight of the reactions released chloride ions, with no free chloride ions in the remaining two. However, because the four free radicals lost electrons at different positions, along with the effects of the substitution positions of -OH and Cl^−^, their activities were different. For example, the radicals formed due to the loss of electrons on the -OH oxygen atom (Figure 15A-①) and the radicals formed by the loss of electrons at the para position on the benzene ring (Figure 15A-④) were more active than the radicals that formed due to the loss of electrons at the ortho position on the benzene ring (Figure 15A-②,③). As shown in Figure 15B-(1), free radicals substituted by chlorine atoms at the ortho position possibly suppressed the reaction due to steric hindrance. Therefore, among the ten reactions, coupling reactions (3) and (8) were most likely to occur, generating two main products: 2,3,6-trichloro-4-(2,3,6-trichlorophenoxy)phenol and 2,2′,3,3′,5,5′-hexachloro-[1,1′-biphenyl]-4,4′-diol (m/z values of both substances are 391.8). Although the chlorine content in 2,3,6-TCP was higher than in 2,6-DCP, the dechlorination efficiency of 2,3,6-TCP converted by LAC-4 was lower than 2,6-DCP. This was attributed to the presence of the chlorine atom at position 3 in 2,3,6-TCP, which prevented the oxidative coupling reaction at position 2.

In summary, LAC-4 first oxidized 2,3,6-TCP into four types of free radicals, and the coupling reaction between the free radicals generated ten types of coupling dimers and released small amounts of chloride ions. Due to the different activities of the free radicals, only two main products were produced: 2,3,6-trichloro-4-(2,3,6-trichlorophenoxy) phenol and 2,2′,3,3′,5,5′-hexachloro-[1,1′-biphenyl]-4,4′-diol.

**Figure 16:**
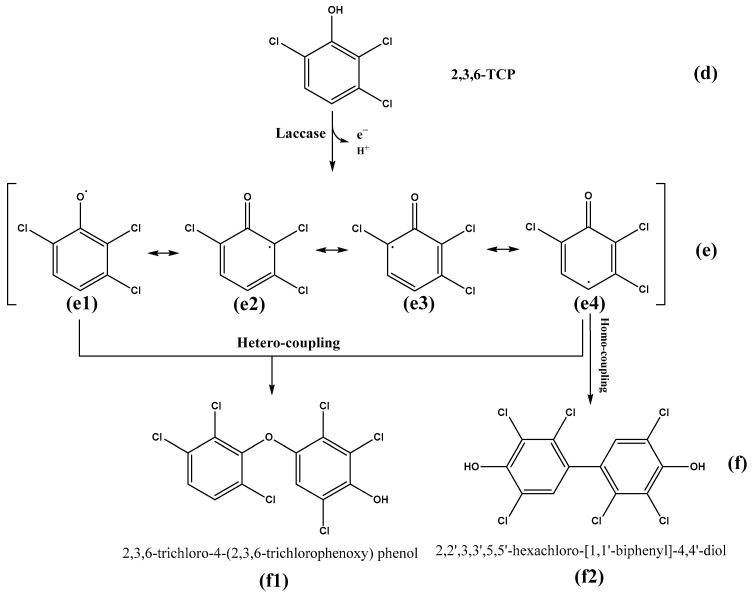
Speculation of the degradation pathway of 2,3,6-TCP catalyzed by LAC-4 laccase. The putative degradation pathway of 2,3,6-TCP catalyzed by LAC-4 laccase.

Figure 16 shows the degradation pathway of LAC-4 laccase, which catalyzed the conversion of 2,3,6-TCP, according to the above results. First, 2,3,6-TCP was oxidized by LAC-4 laccase, causing it to lose electrons and convert into 4 types of free radicals (e1), (e2), (e3), and (e4), and the free radical monomers spontaneously underwent coupling reaction, producing dimer products 2,3,6-trichloro-4-(2,3,6-trichlorophenoxy)phenol and 2,2′,3,3′,5,5′-hexachloro-[1,1′-biphenyl]-4,4′-diol, whose m/z values were 391.8.

Thus, conversion reactions of the two chlorophenols 2,6-DCP and 2,3,6-TCP by LAC-4 laccase shared a common attribute; specifically, the free radicals that formed from the oxidization of 2,6-DCP and 2,3,6-TCP by LAC-4 laccase produced dimers as a result of the polymerization reactions. In summary, LAC-4 catalyzed the polymerization of 2,6-DCP and 2,3,6-TCP, forming dimer products.

#### 3.5.3. 2,6-DCP + 2,3,6-TCP

**Figure 17:**
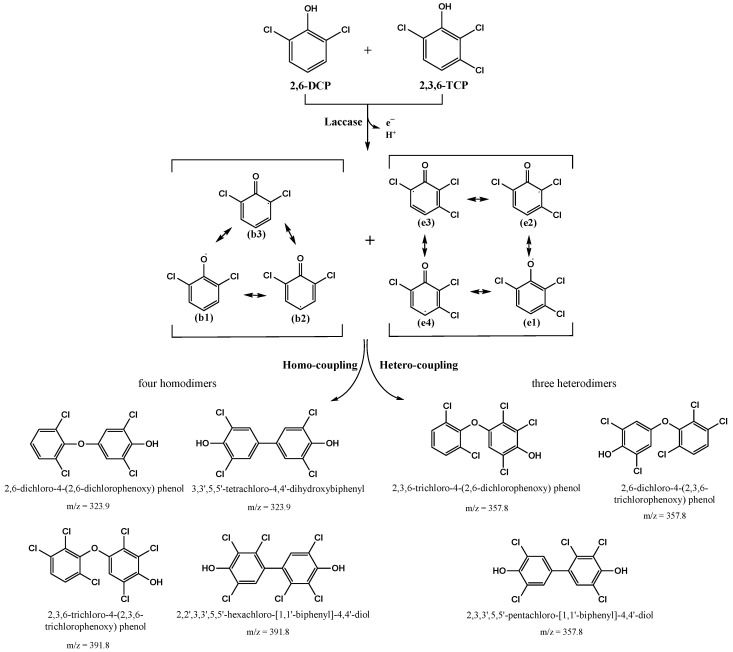
The degradation products of an equivalent mixture of 2,6-DCP and 2,3,6-TCP catalyzed by LAC-4 laccase contain four homodimers and three heterodimers.

We have also performed an experiment to look at degradation of an equivalent mixture of 2,6-DCP and 2,3,6-TCP catalyzed by LAC-4 laccase. We detected the products of LAC-4 laccase degrading a mixture of chlorophenols (2,6-DCP + 2,3,6-TCP, 200 mg/L + 200 mg/L) by GC-MS. The result was shown in Figure 17. As shown in Figure 17, the degradation products of an equivalent mixture of 2,6-DCP and 2,3,6-TCP catalyzed by LAC-4 laccase contain both homodimer and heterodimer metabolites. When an equivalent mixture of 2,6-DCP and 2,3,6-TCP was degraded by LAC-4 laccase, both homodimer and heterodimer metabolites were detected simultaneously.

Homodimer metabolites include the following substances: homopolymeric product of 2,6-DCP (homodimer of 2,6-DCP: 2,6-dichloro-4-(2,6-dichlorophenoxy) phenol, 3,3′,5,5′-tetrachloro-4,4′-dihydroxybiphenyl); homopolymeric product of 2,3,6-TCP (homodimer of 2,3,6-TCP: 2,3,6-trichloro-4-(2,3,6-trichlorophenoxy) phenol, 2,2′,3,3′,5,5′-hexachloro-[1,1′-biphenyl]-4,4′-diol).

Heterodimer metabolites include the following substances: heteropolymeric products of 2,6-DCP and 2,3,6-TCP (heterodimer of 2,6-DCP and 2,3,6-TCP: 2,3,3′,5,5′-pentachloro-[1,1′-biphenyl]-4,4′-diol, 2,3,6-trichloro-4-(2,6-dichlorophenoxy) phenol, 2,6-dichloro-4-(2,3,6-trichlorophenoxy) phenol).

In summary, the degradation products of an equivalent mixture of 2,6-DCP and 2,3,6-TCP catalyzed by LAC-4 laccase contain four homodimers and three heterodimers.

## 4. Discussion

### 4.1. Enzymatic Properties of LAC-4 Laccase

The laccase activity decreased when the temperature exceeded 70 °C. The reasons for the decrease in laccase activity when the temperature exceeds 70 °C are as follows: laccase molecules will unfold with the gradual increase in ambient temperature, and the specific spatial arrangement of peptide chains will also change. In addition, hydrophobic amino acid residues inside laccase molecules will also be exposed. These factors will affect the activity of laccase. When the temperature exceeds 70 °C, the temperature will directly destroy the conformation of laccase and reduce its activity.

The stability of laccase greatly reduced over 30 °C. The reasons that the stability of laccase greatly reduced over 30 °C are as follows: Previous studies have shown that when the temperature exceeds 30 °C, although the secondary structure has not been destroyed, the peptide chain of laccase is induced to extend. Laccase changes from tightly ordered structure to loose conformation. Therefore, the stability of laccase significantly decreased over 30 °C.

In this study, we found that LAC-4 had poor stability under alkaline conditions. When the pH values were 9.0 and 10.0, the remaining activities after 4 h were only 4.05% and 4.24%, respectively. The reason for this phenomenon is described as follows. Under alkaline conditions, OH^−^ can enter the active site of laccase and inhibit the transfer of electrons from the T1 site to the T2/T3 site (the center of the trinuclear copper cluster), thereby inhibiting the activity of laccase. An alkaline environment can also deprotonate amino acids in the O_2_ pocket of laccase [36]. In summary, under alkaline conditions, OH^−^ directly acts on the active center of laccase, blocking electron transfer and deprotonating amino acid residues in the active center. The above factors lead to the significant decrease in laccase stability under alkaline conditions.

In this study, we found that LAC-4 showed good stability in organic solvents such as propylene glycol, ethylene glycol, and glycerol. The reason for this phenomenon is described as follows. 1,2-propanediol, ethylene glycol and glycerol all contain two or more hydroxyl groups. Previous studies have shown that the interaction between polyhydroxy compounds and laccase can weaken the adverse effect of temperature on laccase. The addition of polyhydroxy compounds changed the microenvironment of amino acid residues of laccase. Moreover, polyhydroxy compounds can increase the content of β-sheet in laccase protein, while reducing the content of random coil, thereby improving the stability of laccase. In addition, the hydroxyl groups in polyhydroxy compounds may also form hydrogen bonds with laccase molecules, further improving the stability of laccase [37].

In this study, we found that ethanol considerably reduced the stability of LAC-4 laccase. After LAC-4 was incubated for 12 h in a solution containing 50% ethanol, laccase activity decreased to 14.02% of the initial enzyme activity, and after 24 h of incubation, the LAC-4 laccase activity essentially disappeared. The reason for this phenomenon is described as follows. The study by Jafari et al. showed that 50% ethanol had a considerable effect on the secondary structure (β-sheet and β-turn) of laccase, which may negatively affect the native structure of laccase. Moreover, salt bridges on the protein surface also play a crucial role in stabilizing the tertiary structure of globular proteins. The salt bridges exposed to ethanol are weak or unstable, so ethanol may affect the structural integrity of the laccase by weakening the salt bridges on the surface of the laccase. In addition, ethanol can remove very important water molecules from the hydrated shell of biocatalysts, and the changes of important water molecules in the hydrated shell may inactivate proteins [38].

In this study, we also found that acetonitrile significantly affected the stability of LAC-4 laccase. Acetonitrile could considerably reduce the stability of LAC-4 laccase. The reason for this phenomenon is described as follows. As a higher polarity organic solvent, acetonitrile can quickly penetrate into the hydrophobic core of the protein, changing the hydrodynamics near the catalytic site, and water is very important for the binding of enzyme to substrate. Acetonitrile can also form a strong hydrogen bond interaction with the amino acid residues at the catalytic site of laccase, which affects the catalysis of the substrate. In addition, acetonitrile may also change the tertiary structure of laccase, resulting in the decrease in laccase stability [39].

### 4.2. Degradation Mechanism of Chlorophenols by LAC-4

By comparing the ability of LAC-4 to degrade three different structures of chlorophenols, it was shown that LAC-4 presented the highest ability to degrade 2,6-DCP, followed by 2,3,6-TCP and 3-CP. This result is consistent with the study reported by Kadhim et al. [29]. In their study, they used the laccase from *Coriolus versicolor* to degrade different chlorophenols, and showed that laccase had a lower catalytic efficiency for meta-substituted compounds. The degradation ability of chlorophenol is likely to be closely related to the number and position of chlorine substituents in the benzene ring. Compared with meta-substitution, ortho-substitution is more likely to be oxidized [29]. 2,6-DCP is more easily degraded than 2,3,6-TCP and 3-CP, because two chlorine atoms are located adjacent to the hydroxyl group, and 2,6-DCP has one less chlorine atom than 2,3,6-TCP, and the slight steric hindrance may also be a reason for the higher degradation rate of 2,6-DCP than 2,3,6-TCP. 3-CP contains fewer chlorine substituents than 2,3,6-TCP, but the degradation rate is lower. It is postulated that the chlorine atom substituted at the 3′ position in the benzene ring is difficult to oxidize, while 2,3,6-TCP oxidation occurs in the adjacent position (position 2 and 6).

Kadhim et al. studied the effect of different substitution positions of chlorine atoms on degradation. The mono-chlorophenol degradation results showed that laccase had a low ability to degrade meta-substituted chlorophenol compared to the ortho and para positions, and only 23% could be removed in 24 h. With respect to dichlorophenol, 2,4-dichlorophenol is the easiest to remove. The removal efficiency of 3,4-dichlorophenol and 2,5-dichlorophenol with one meta substituent is approximately the same. The removal efficiency of 3,5-dichlorophenol with two meta substitution sites is the lowest. For trichlorophenol, the easiest compound to remove is 2,4,6-TCP, occupying only the ortho and para positions on the benzene ring, while the removal of 2,4,5-TCP and 2,3,6-TCP showed low efficiency [29].

During the degradation of 2,6-DCP and 2,3,6-TCP by LAC-4, the presence of free chloride ions was observed. The release of chloride ions is not mediated by laccase but is caused by the oxidative coupling of free radicals, which was proved in the study of Dec et al. [30], who showed that dechlorination involved free radical oxidation coupling [30]. LAC-4 catalyzes 2,6-DCP to form three free radicals; however, only two non-dechlorinated dimers were detected by GC-MC. The underlying reason could be that the free radicals of lone pair electrons in oxygen and para forms are more active and more likely to react. This results in O-para, O-O, and para-para couplings taking priority over other couplings. The peroxide formed by O-O coupling is very unstable, and most of the reaction products are thus O-para and para-para. A small amount of O-ortho, para-ortho and o-ortho-coupling releases chloride ions. This explains that only a small amount of free chloride ions were present during the degradation of chlorophenol by LAC-4. This result is consistent with the result of LAC-4 catalyzing 2,6-DCP to release only a small amount of chloride ions.

Research performed by Dec et al. showed that laccase could catalyze 2,4-dichlorophenol to form four types of free radicals, and the coupling reactions between free radicals yielded ten types of dimers, of which seven types of reactions could release chloride ions [30]. In this study, it was found that 2,6-dichlorophenol catalyzed by LAC-4 could generate three stable free radicals, and six similar coupling reactions occurred. The two main products produced were identified by GC-MS analysis. As shown in Figure S4, 2,6-DCP (a) loses an electron under the catalysis of LAC-4 and is converted into three free radicals (B1), (B2) and (B3). Two main products (C1) and (C2) that can be detected by GC-MS are generated by polymerization between free radicals. However, it is also possible to produce four other polymers that have not been detected: (C3), (C4), (C5) and (C6). The dechlorination study of LAC-4 degradation of 2,6-DCP showed that a small amount of chloride ions were released during the degradation process. This result indicates that several other polymerization reactions with chloride ion release may occur, resulting in the formation of (C3)-(C6). However, because the generated amount is too small, it cannot be detected by GC-MS.

Currently, there are very few studies on the structural identification of polymers produced by laccase-catalyzed polymerization of chlorophenol. This study is the first to analyze and identify the chemical structures of two polymers produced by 2,6-dichlorophenol and 2,3,6-trichlorophenol catalyzed by LAC-4 laccase. The degradation pathway of 2,6-dichlorophenol and 2,3,6-trichlorophenol catalyzed by LAC-4 has been investigated. To our knowledge, our study revealed the transformation pathway of 2,6-dichlorophenol and 2,3,6-trichlorophenol catalyzed by laccase for the first time, which was also one of the main innovations of this study. The results of this study showed high theoretical significance for further revealing the reaction mechanism of laccase catalysis of chlorophenol, which could provide new clues and perspectives.

### 4.3. Tolerance of LAC-4 for Different Metal Salts and Organic Solvents during Degradation of the Two Chlorophenols

This study found that LAC-4 laccase degradation of chlorophenol has a strong tolerance to Mn^2+^, Mg^2+^, Na^+^, Cu^2+^, and K^+^. Different metal ions can affect laccase activity in different ways. Na^+^ and K^+^ can dehydrate the hydrophobic regions of laccase protein, improve the internal interaction of laccase, and thus enhance the stability of laccase [40]. Mn^2+^ can improve the activity of acid protease, mainly because Mn^2+^ contributes to the stability of the acid protease molecular structure and prevents its conformation change during the catalytic reaction, thereby improving the reaction efficiency. Hence, Mn^2+^ is often used as a stabilizer for acid protease [41]. The optimal reaction pH of LAC-4 laccase is in the acidic range. Laccase may have similar properties to acidic protease. Therefore, Mn^2+^ can also stabilize the molecule structure of the laccase, making laccase more resistant to Mn^2+^ during the degradation of chlorophenol. Mg^2+^ is a catalytic activator of many oxidoreductases, and laccase is also an oxidoreductase, which may explain why laccase is more resistant to Mg^2+^ during the degradation of chlorophenols. Laccase has a strong tolerance to Cu^2+^ during degrading chlorophenol, which may be related to the fact that laccase contains 4 Cu^2+^, and Cu^2+^ plays an important role in the catalytic reaction of laccase.

Research on the tolerance of laccase to different organic solvents helps us to screen out more effective cosolvents, while organic solvents have less effect on laccase activity and enhance the solubility of chlorophenol in the reaction solution, thereby enhancing the ability of laccase to degrade chlorophenol. In this study, LAC-4 showed a strong tolerance for ethylene glycol and glycerol for the degradation of 2,6-DCP and 2,3,6-TCP. The results of the effects of different organic solvents on LAC-4 laccase activity suggested that Lac-4 had the strongest tolerance to ethylene glycol and glycerol. Thus, the strong tolerance of laccase activity to ethylene glycol and glycerol is an important reason for the strong tolerance of LAC-4 laccase to ethylene glycol and glycerol during the degradation of chlorophenols. However, we found that acetonitrile has the strongest inhibitory effect on the degradation of 2,6-DCP and 2,3,6-TCP by LAC-4. The protein molecules in the solution are surrounded by a hydrated shell, which consists of water molecules attached to the surface of the protein mainly through hydrogen bonding, and is essential to support the natural protein conformation. If an organic solvent is present in the solution, its molecules tend to remove water from the hydrated shell, thereby disrupting the interaction responsible for maintaining a fine balance of the protein molecule’s natural conformation. The destruction of the hydrated shell is one of the main causes of denaturation of proteins by organic solvents [42,43]. In comparison with other organic solvents, acetonitrile has a stronger destructive effect on the hydrated shell. In addition, acetonitrile may also combine with the catalytic active site of laccase protein while destroying the hydrated shell, thereby strongly inhibiting the degradation of chlorophenol by laccase.

### 4.4. Detoxification of Chlorophenols 2,6-DCP and 2,3,6-TCP by LAC-4

In this study, the toxicity of LAC-4 degradation products 2,6-DCP and 2,3,6-TCP was determined by detecting the germination and growth status of three plant seeds. The results showed that the germination rate of the plant seeds and bud and root growth in the chlorophenol solution after LAC-4 treatment were greatly improved, indicating that LAC-4 has a good detoxification effect on 2,6-DCP and 2,3,6-TCP. LAC-4 can not only efficiently degrade 2,6-dichlorophenol and 2,3,6-trichlorophenol, but also reduce or even completely eliminate the toxicity of chlorophenols. However, previous researcher used *E. coli* capable of expressing recombinant GFP to evaluate the toxicity of the degradation products of photocatalysis and laccase oxidation of PCP and 2,4-DCP and found that the toxicity of the laccase-catalyzed chlorophenol reaction solution was more obvious and durable [33]. This indicates that the difference in the structure of chlorophenols and the different ways of treating chlorophenols may lead to different detoxification effects of laccase on chlorophenols.

This study found that LAC-4 can completely eliminate the toxicity of 100 mg/L and 200 mg/L 2,3,6-trichlorophenol. However, when the concentration of chlorophenol was higher, even if the degradation rate reached 100%, LAC-4 could only reduce the toxicity of chlorophenol without completely eliminating the toxicity. The same situation also occurred in the degradation of 2,6-dichlorophenol by LAC-4. The reason may be that toxic by-products were generated during the degradation process. A small amount of toxic by-products were produced when the concentration of chlorophenol was low, so no toxic effects on the plant seeds were detected, while a large amount of toxic by-products were produced when the concentration of chlorophenol was high, thereby showing a strong toxic effect.

For both mung bean and wheat seeds, 2,3,6-trichlorophenol with a concentration of 400 mg/L had higher toxicity than 2,6-dichlorophenol because the toxicity of chlorophenol increases as the number of chlorine substituents increases. At an equivalent degradation rate, the detoxification rate of 2,3,6-trichlorophenol by LAC-4 was higher than that of 2,6-dichlorophenol. For example, for wheat seeds, the detoxification rate of 2,3,6-trichlorophenol was two times that of 2,6-dichlorophenol. This result shows that LAC-4 has a better detoxification effect on the more toxic chlorophenol, which is also a unique feature and advantage of LAC-4 in detoxifying chlorophenol. Previous studies have found that the toxicity of chlorophenol degradation products is greater than the original chlorophenol, and two methods, photocatalysis and laccase catalysis, are used to synergistically treat chlorophenol, resulting in a more toxic chemical than pentachlorophenol and 2,4-dichlorophenol. The substance may have formed by-products, including dioxins or dibenzofurans during the catalysis process [33].

LAC-4 has a good detoxification effect on 2,6-dichlorophenol and 2,3,6-trichlorophenol, because LAC-4 catalyzes the polymerization of 2,6-dichlorophenol and 2,3,6-trichlorophenol, forming insoluble precipitates through the polymerization reaction, thereby reducing the phytotoxicity of 2,6-dichlorophenol and 2,3,6-trichlorophenol. In addition, 2,6-dichlorophenol and 2,3,6-trichlorophenol catalyzed by laccase can cause dechlorination, and the dechlorination products usually have minimal toxicity. Thus, dechlorination can reduce the toxicity of chlorophenols.

## 5. Conclusions

The enzymatic properties of the laccase LAC-4 purified from *Ganoderma lucidum* were systematically studied. LAC-4 had the best stability under neutral pH 7.0 conditions. LAC-4 stability under neutral and acidic conditions was considerably higher than under alkaline conditions. LAC-4 exhibited a strong tolerance for inhibitors such as EDTA-2Na and SDS, metal salts such as MnSO_4_, ZnSO_4_, MgSO_4_, Na_2_SO_4_, CuSO_4_, and organic solvents such as ethylene glycol and glycerol. LAC-4 also showed a strong stability to metal salts such as CaCl_2_, MgCl_2_, NaCl, KCl, and LiCl, organic solvents such as DMSO, 1,2-propanediol and ethylene glycol. High concentrations of DMSO could improve the stability of LAC-4 laccase. The degradation, detoxification and degradation mechanism of LAC-4 laccase on chlorophenols with different structures were further studied. LAC-4 had a strong ability for 2,6-DCP and 2,3,6-TCP degradation. LAC-4 also had a good degradation effect on the chlorophenol mixture (2,6-DCP + 2,3,6-TCP). The catalytic efficiency and the catalytic rate of LAC-4 on 2,6-DCP were significantly higher than 2,3,6-TCP. LAC-4 had a strong tolerance for high concentrations of different metal salts and organic solvents during degradation of 2,6-DCP and 2,3,6-TCP. LAC-4 treatment had a good detoxification effect on the phytotoxicity of individual chlorophenols (2,6-DCP, 2,3,6-TCP) and chlorophenol mixtures (2,6-DCP + 2,3,6-TCP). Treatment with LAC-4 significantly reduced or completely eliminated individual chlorophenol and chlorophenol mixture toxicities on the three types of plant seeds. The degradation mechanisms and pathways of 2,6-DCP and 2,3,6-TCP by laccase were revealed for the first time. The putative degradation pathway of 2,6-DCP and 2,3,6-TCP catalyzed by LAC-4 was obtained. LAC-4 catalyzed the polymerization of 2,6-DCP and 2,3,6-TCP, forming dimer products. LAC-4 catalyzed 2,6-DCP into two main products: 2,6-dichloro-4-(2,6-dichlorophenoxy) phenol and 3,3′,5,5′-tetrachloro-4,4′-dihydroxybiphenyl. LAC-4 catalyzed 2,3,6-TCP into two main products: 2,3,6-trichloro-4-(2,3,6-trichlorophenoxy)phenol and 2,2′,3,3′,5,5′-hexachloro-[1,1′-biphenyl]-4,4′-diol. Taken together, LAC-4 laccase has great practical value and application potential in the treatment of chlorophenol wastewater or the remediation of an environment contaminated by chlorophenol.

## Figures and Tables

**Table 1:** The kinetic parameters of purified LAC-4 laccase.

	K_m_ (mM)	V_max_ (mM/s)	K_cat_ (s^−1^)	K_cat_/K_m_ (s^−1^mM^−1^)
ABTS	0.075	3.988 × 10^−7^	9.42	126.27
2,6-DMP	0.803	1.085 × 10^−7^	2.56	3.19
Guaiacol	0.097	1.906 × 10^−7^	4.5	46.34

**Table 2:** The kinetic parameters of degradation of 2,6-DCP and 2,3,6-TCP by LAC-4.

	K_m_ (mM)	V_max_ (mM/s)	K_cat_ (s^−1^)	K_cat_/K_m_ (s^−1^mM^−1^)
2,6-DCP	3.636	2.74 × 10^−3^	1.23	0.338
2,3,6-TCP	4.875	1.50 × 10^−3^	0.67	0.137

**Table 3:** Effects of different concentrations of 2,6-DCP on the germination rates of wheat seeds before and after degradation by LAC-4 laccase.

	2,6-DCP								
	Control	100-0	100-1	200-0	200-1	400-0	400-1	600-0	600-1
wheat seed germination rate (%)	95	95	95	80	95	60	100	0	80

Control: no addition of 2,6-DCP; 100-0: 100 mg/L of 2,6-DCP not treated with LAC-4; 100-1: 100 mg/L of 2,6-DCP treated with LAC-4; 200-0: 200 mg/L of 2,6-DCP not treated with LAC-4; 200-1: 200 mg/L of 2,6-DCP treated with LAC-4; 400-0: 400 mg/L of 2,6-DCP not treated with LAC-4; 400-1: 400 mg/L of 2,6-DCP treated with LAC-4; 600-0: 600 mg/L of 2,6-DCP not treated with LAC-4; 600-1: 600 mg/L of 2,6-DCP treated with LAC-4.

**Table 4:** Effects of different concentrations of 2,6-DCP on the germination rates of rice seeds before and after degradation by LAC-4 laccase.

	2,6-DCP				
	Control	400-0	400-1	600-0	600-1
rice seed germination rate (%)	95	70	85	0	50

Control: no addition of 2,6-DCP; 400-0: 400 mg/L of 2,6-DCP not treated with LAC-4; 400-1: 400 mg/L of 2,6-DCP treated with LAC-4; 600-0: 600 mg/L of 2,6-DCP not treated with LAC-4; 600-1: 600 mg/L of 2,6-DCP treated with LAC-4.

**Table 5:** Effects of different concentrations of 2,3,6-TCP on the germination rates of wheat and rice seeds before and after degradation by LAC-4 laccase.

	2,3,6-TCP						
	Control	100-0	100-1	200-0	200-1	400-0	400-1
wheat seed germination rate (%)	90	35	95	15	95	0	25
rice seed germination rate (%)	95	75	80	0	80	0	50

Control: no addition of 2,3,6-TCP; 100-0: 100 mg/L of 2,3,6-TCP not treated with LAC-4; 100-1: 100 mg/L of 2,3,6-TCP treated with LAC-4; 200-0: 200 mg/L of 2,3,6-TCP not treated with LAC-4; 200-1: 200 mg/L of 2,3,6-TCP treated with LAC-4; 400-0: 400 mg/L of 2,3,6-TCP not treated with LAC-4; 400-1: 400 mg/L of 2,3,6-TCP treated with LAC-4.

**Table 6:** Effects of different concentrations of mixed chlorophenol (2,6-DCP + 2,3,6-TCP) on the germination rates of mung bean and wheat seeds before and after degradation by LAC-4 laccase.

	2,6-DCP + 2,3,6-TCP				
	Control	100-0	100-1	200-0	200-1
mung bean seed germination rate (%)	100	100	100	70	100
wheat seed germination rate (%)	95	40	90	0	15

Control: no addition of mixed chlorophenols; 100-0: mixed chlorophenols (100 mg/L 2,6-DCP + 100 mg/L 2,3,6-TCP) not treated with LAC-4; 100-1: mixed chlorophenols (100 mg/L 2,6-DCP + 100 mg/L 2,3,6-TCP) treated with LAC-4; 200-0: mixed chlorophenols (200 mg/L 2,6-DCP + 200 mg/L 2,3,6-TCP) not treated with LAC-4; 200-1: mixed chlorophenols (200 mg/L 2,6-DCP + 200 mg/L 2,3,6-TCP) treated with LAC-4.

## Supplementary Materials

The following supporting information can be downloaded at: https://www.mdpi.com/article/10.3390/ijerph19138150/s1, Figure S1: Effects of different concentrations of metal salts on the degradation of 2,3,6-TCP by LAC-4; Figure S2: Effects of 11 organic solvents at different concentrations on the degradation of 2,3,6-TCP by LAC-4; Figure S3: Detection of the dechlorination of 2,6-DCP and 2,3,6-TCP by LAC-4 using the silver nitrate turbidimetry method; Figure S4: The putative transformation pathway of 2,6-DCP catalyzed by LAC-4 laccase.
