# Supplementary figures and images for: Degradation and Detoxification of Chlorophenols with Different Structure by LAC-4 Laccase Purified from White-Rot Fungus Ganoderma lucidum
Source: Int J Environ Res Public Health. 2022 Jul 2;19(13):8150. doi: 10.3390/ijerph19138150 (PMC9266351; doi:10.3390/ijerph19138150)

**A** Fig.S1

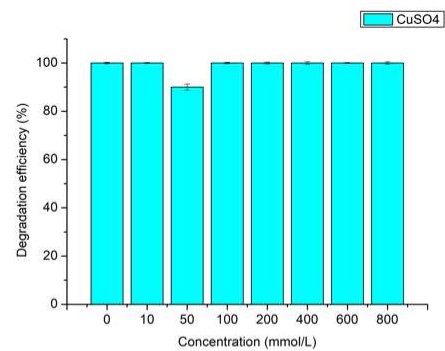

**B**

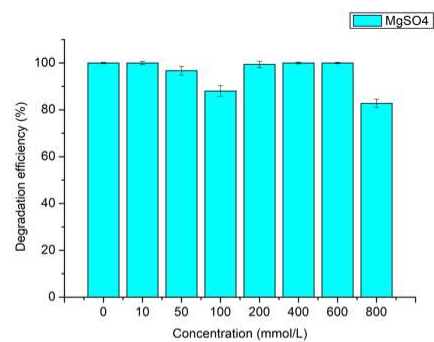

**C**

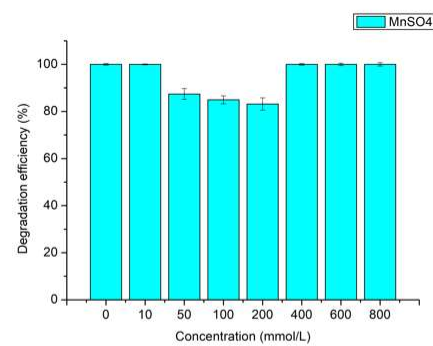

**D**

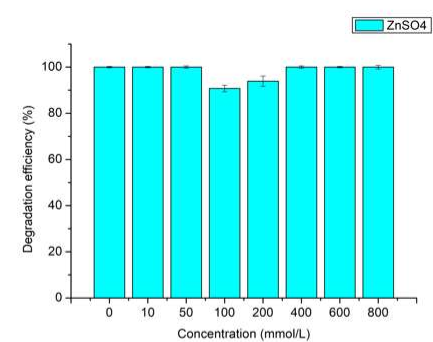

**E**

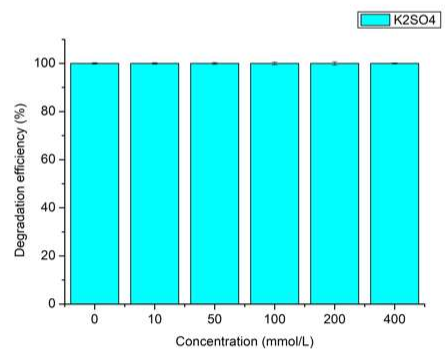

**F**

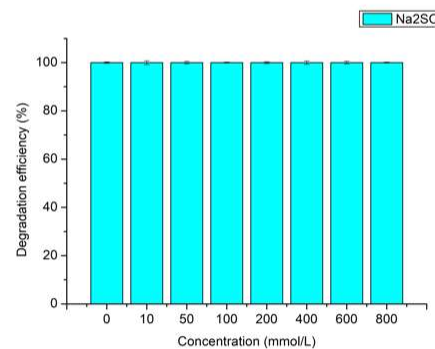

**G**

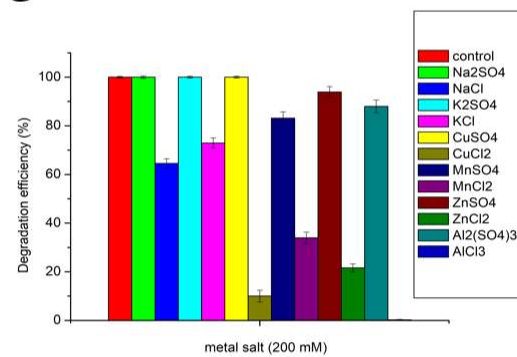

Supplement: Supplementary file 1 [file ijerph-19-08150-s001.zip › Fig.S1-2021.12.6.pdf]

**A** Fig.S2

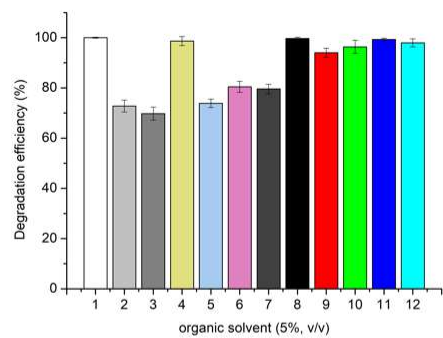

**B**

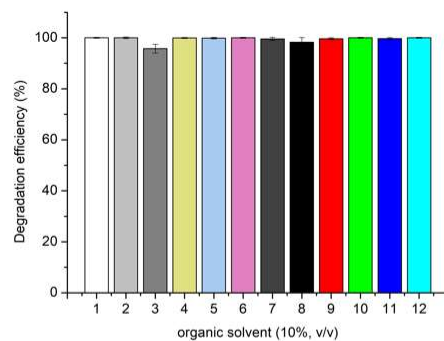

**C**

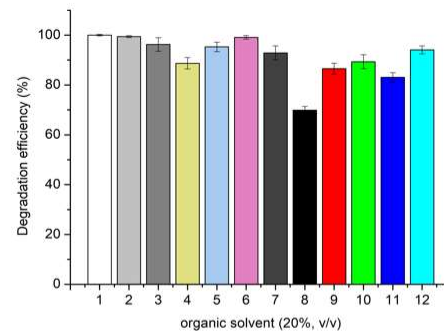

**D**

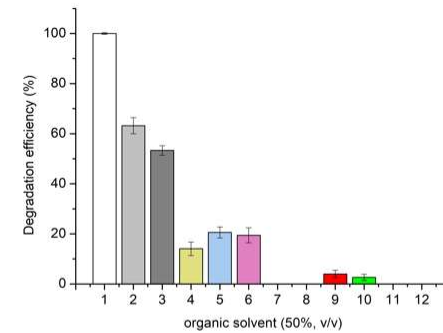

**E**

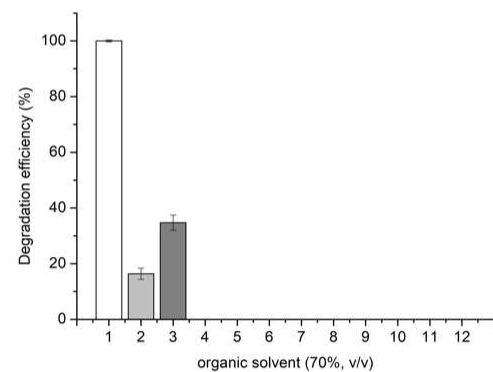

Supplement: Supplementary file 1 [file ijerph-19-08150-s001.zip › Fig.S2-2021.12.6.pdf]

Fig.S3

**A**

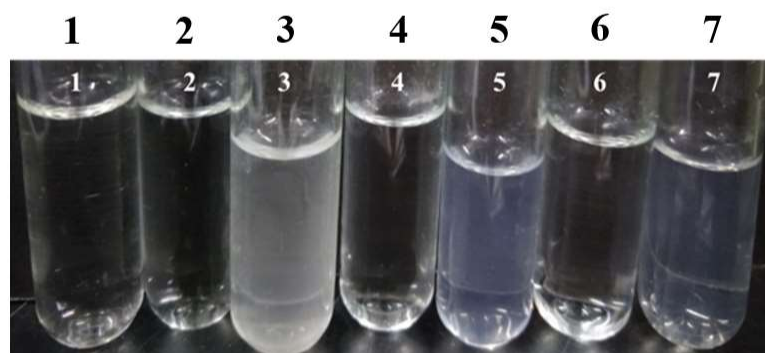

**B**

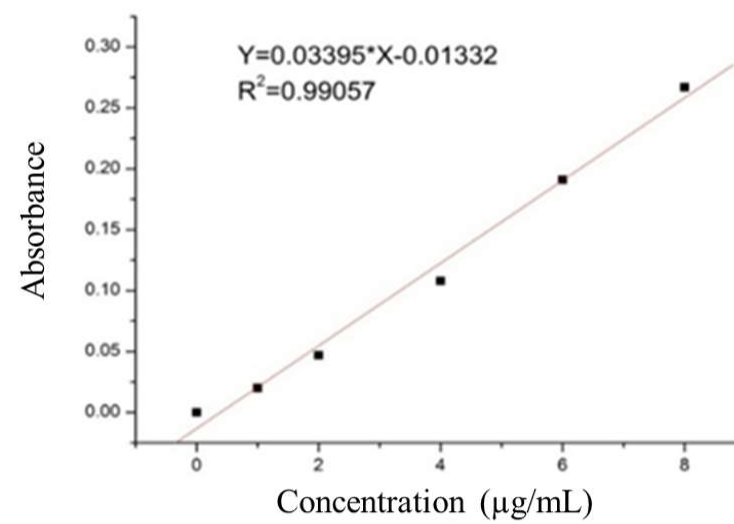

Supplement: Supplementary file 1 [file ijerph-19-08150-s001.zip › Fig.S3-2021.12.6.pdf]

Fig.S4

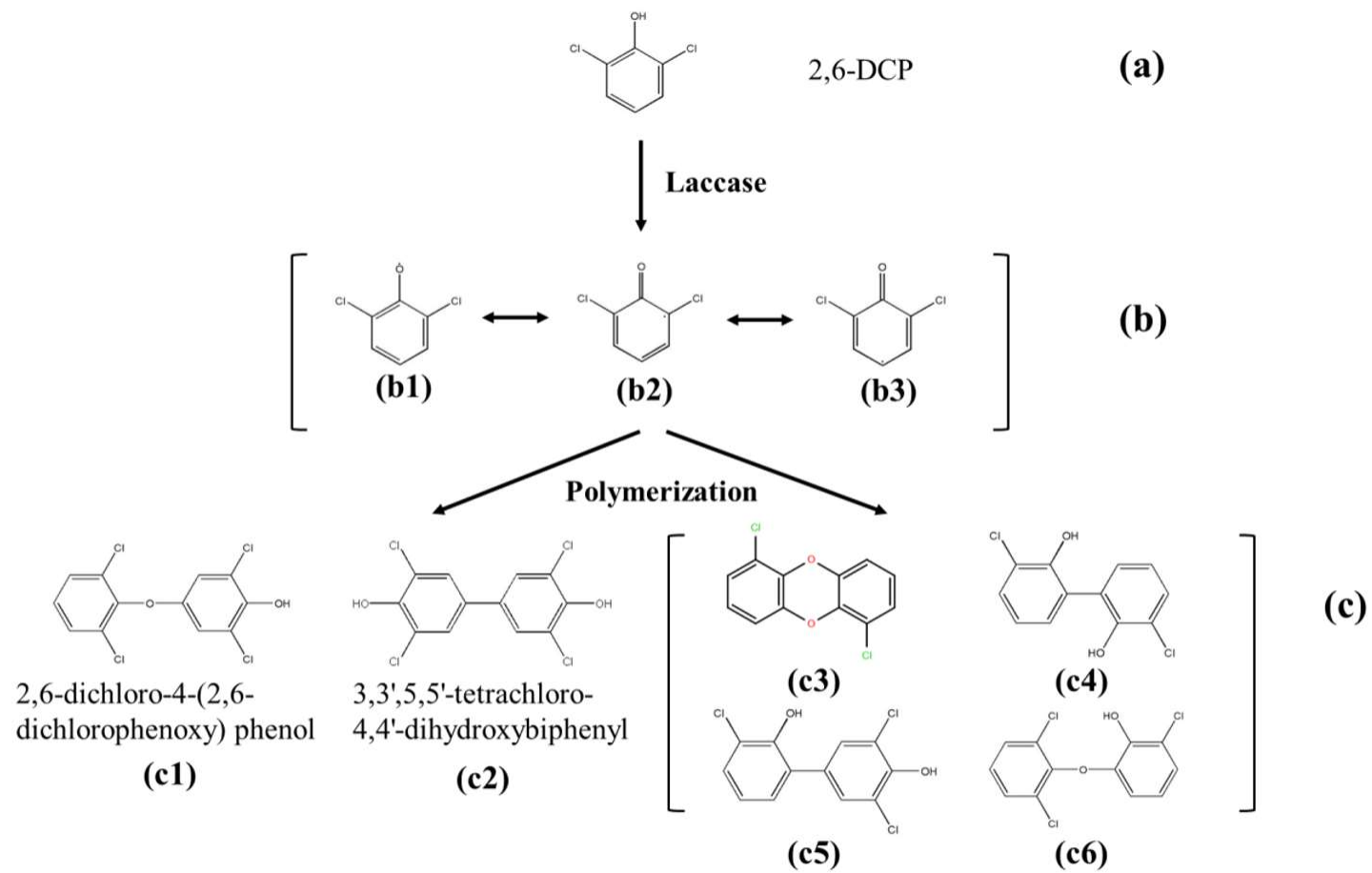

Supplement: Supplementary file 1 [file ijerph-19-08150-s001.zip › Fig.S4-2021.12.6.pdf]
